# Entropy Monotonicity and Superstable Cycles for the Quadratic Family Revisited

**DOI:** 10.3390/e22101136

**Published:** 2020-10-07

**Authors:** José M. Amigó, Ángel Giménez

**Affiliations:** Centro de Investigación Operativa, Universidad Miguel Hernández, Avda. de la Universidad s/n, 03202 Elche, Spain; a.gimenez@umh.es

**Keywords:** topological entropy, quadratic maps, Milnor’s Monotonicity Conjecture, superstable cycles, root branches, transversality

## Abstract

The main result of this paper is a proof using real analysis of the monotonicity of the topological entropy for the family of quadratic maps, sometimes called Milnor’s Monotonicity Conjecture. In contrast, the existing proofs rely in one way or another on complex analysis. Our proof is based on tools and algorithms previously developed by the authors and collaborators to compute the topological entropy of multimodal maps. Specifically, we use the number of transverse intersections of the map iterations with the so-called critical line. The approach is technically simple and geometrical. The same approach is also used to briefly revisit the superstable cycles of the quadratic maps, since both topics are closely related.

## 1. Introduction

Topological entropy is one of the main quantifiers of complexity in continuous dynamics. First of all, it is a tight upper bound of all measure-preserving dynamics generated by a given continuous self-map of a compact metric space [1]. Furthermore, in metric spaces, topological entropy measures the growth rate of the number of ever longer orbits up to a small error [2]. Its analytical calculation is only feasible in some special cases, though. For one-dimensional dynamics, where transformations can be supposed to be continuous and piecewise monotone (multimodal) for practical purposes, a number of numerical algorithms based on symbolic representations of the orbits have been developed. Examples include kneading invariants [3], min-max symbols [4], ordinal patterns [5], context trees [6] and more. Precisely, this paper is the outgrowth of previous work by the authors and collaborators on the numerical computation of the topological entropy of multimodal maps using min-max symbols [7,8,9]. At the heart of our algorithms is the number of transverse intersections (i.e., “X-crossings”) of a multimodal map and its iterates with the so-called critical lines. In this paper we also show the potential of this concept in regard to theoretical issues. To this end, we revisit two well-traveled topics in one-dimensional dynamics:

**(i)**  The monotonicity of the topological entropy for the family of quadratic maps;
**(ii)**  Some basic properties of the periodic orbits of its critical point (superstable cycles).

Next, we elaborate a bit on these two topics.

The family of quadratic maps (or quadratic family) is composed of the logistic maps $f_{\mu }\left(x\right)=4\mu x\left(1-x\right)$, 0≤μ≤1, or, for that matter, any other dynamically equivalent maps; actually, we will use the maps $q_{t}\left(x\right)=t-x^{2}$, 0≤t≤2, because they are algebraically handier. When the topological entropy of multimodal maps was studied in the 1980s, the numerical results indicated that the topological entropy of the quadratic family was a monotone function of the parameter. This property entered the literature as Milnor’s Monotonicity Conjecture, although what he actually conjectured was the connectivity of the isentropes (i.e., the sets of parameters for which the topological entropy is constant) of the cubic maps in [10], when the monotonicity of the topological entropy for the quadratic family had already been proved by himself (in collaboration with W. Thurston) [3] as well as by other authors [11,12,13]. According to [14,15], all these proofs use that the quadratic map can be extended to the complex plane and require tools from complex analysis. At variance, the proof of Milnor’s Monotonicity Conjecture presented in this paper (Section 4, Theorem 2) uses only real analysis. The conjecture was later generalized to multimodal maps and was recently proved in [15].

Points where a multimodal map achieves its local extrema are generically called critical points (also when the map is not differentiable there); for example, the critical point of $f_{\mu }$ is x=1/2 for all μ, while the critical point of $q_{t}$ is x=0 for all *t*. Orbits generated by any of the critical points play an important role not only in symbolic dynamics (via, e.g., the kneading invariants) but also in the stability of fixed points and periodic attractors. Thus, unimodal maps with a negative Schwarzian derivative (except at the critical point) and an invariant boundary, such as the quadratic maps, have at most one stable periodic orbit, namely, the one (if any) whose attraction basin contains the critical point [16,17]; these are the periodic attractors that can be seen in the bifurcation diagram (Section 3). On the other hand, if the critical point of a quadratic map is eventually periodic, then the periodic cycle is unstable [11].

However, we will only touch upon stability in passing. The reason why we include the superstable cycles of the quadratic maps in this paper is two-fold. First and least, the mathematical techniques used to deal with both topics, entropy monotonicity and superstable cycles, are similar, so we can easily exploit this fact. More importantly, the relationship between these topics is deeper than might be thought. Indeed, Thurston’s Rigidity, a result on the periodic orbits of the critical points of quadratic maps, implies Milnor’s Monotonicity Conjecture for the quadratic maps and it is necessary (in a generalized version) to prove the case of polynomial maps of higher degrees [15]. Here we will give only a general idea of this relationship. In addition, we will briefly discuss the “dark lines” through the chaotic bands of the bifurcation diagram of the quadratic family, which relate to the orbit of the critical point, as well as the parameter values for which the critical point is eventually periodic (Misiurewicz points).

This paper is organized as follows. In Section 2 we introduce the mathematical background needed for the following sections. In particular, we introduce the expression of the topological entropy for multimodal maps via the number of transverse crossings of its iterates with the critical lines. More specific concepts and tools that refer to the family of the quadratics maps (Section 3) are discussed in Section 3.1 (root branches) and Section 3.2 (smoothness domains of the root branches). Root branches have many interesting properties but we only address those we need for our purposes. The materials of Section 2 and Section 3 will then be used in two complementary ways. The proof in Section 4 that the smoothness domains of the root branches are half-intervals lead to the monotonicity of the topological entropy for the quadratic family. The bifurcation points of some root branches lead to the basic properties and parameter values of the superstable cycles of the quadratic family (Section 5.1). The latter topic will be completed with a short digression on the eventually periodic orbits of the critical point (Section 5.2).

## 2. Mathematical Preliminaries

### 2.1. Multimodal Maps

Let *I* be a compact interval [a,b]⊂R and f:I→I be a piecewise monotone continuous map. Such a map is called *l*-modal if *f* has local extrema at precisely *l* interior points $c_{1}<\ldots <c_{l}$. Moreover, we assume that *f* is strictly monotone in each of the l+1 intervals

$$I_{1}=\left[a,c_{1}\right),I_{2}=\left(c_{1},c_{2}\right),\ldots ,I_{l}=\left(c_{l-1},c_{l}\right),I_{l+1}=\left(c_{l},b\right].$$

The points $c_{1},\ldots ,c_{l}$ are called critical or turning points and their images $f(c_{1})$, …, $f(c_{l})$ are the critical values of *f*. These maps are also referred to as multimodal maps (for a general *l*) and unimodal maps (if l=1). We denote the set of *l*-modal maps by $M_{l}\left(I\right)$, or just $M_{l}$ if the interval *I* is clear from the context or unimportant for the argument. $f\in M_{l}\left(I\right)$ is said to have positive (resp. negative) shape if $f(c_{1}$) is a maximum (resp. minimum); here and hereafter, all extrema are meant to be local unless stated otherwise. Thus, if *f* has positive shape, then *f* is strictly increasing in the intervals with odd subindex ($I_{\mathrm{odd}}$) and strictly decreasing in the intervals with even subindices ($I_{\mathrm{even}}$).

For n≥0, $f^{n}$ denotes the *n*th iterate of *f*, where $f^{0}$ is the identity map. Since *f* is continuous and piecewise strictly monotone, so is $f^{n}$ for all n≥1. The proof of the following Proposition is direct (see [8], Lemma 2.2).

**Proposition** **1.** *Let $f\in M_{l}\left(I\right)$ with positive shape and n≥1. We have:*

(1) $$f^{n+1}\left(x\right)\;is\;a\;maximum\;if\;\left\{\begin{array}{c} \left(i\right)\;f^{n}\left(x\right)=c_{odd},\\ \left(ii\right)\;f^{n}\left(x\right)\in I_{even}\;and\;f^{n}\left(x\right)\;is\;a\;minimum,\;or\\ \left(iii\right)\;f^{n}\left(x\right)\in I_{odd}\;and\;f^{n}\left(x\right)\;is\;a\;maximum, \end{array}\right.$$

*and*

(2) $$f^{n+1}\left(x\right)\;is\;a\;minimum\;if\;\left\{\begin{array}{c} \left(i\right)\;f^{n}\left(x\right)=c_{even},\\ \left(ii\right)\;f^{n}\left(x\right)\in I_{odd}\;and\;f^{n}\left(x\right)\;is\;a\;minimum,\;or\\ \left(iii\right)\;f^{n}\left(x\right)\in I_{even}\;and\;f^{n}\left(x\right)\;is\;a\;maximum. \end{array}\right.$$

If *f* has negative shape, then replace “$f^{n+1}\left(x\right)$ is a maximum if” by “$f^{n+1}\left(x\right)$ is a minimum if” in (1), and the other way around in (2).

Apply Proposition 1 to $f^{n}$, $f^{n-1}$, …, *f* to conclude that $f^{n+1}$ has local extrema at all x∈I such that $f^{k}\left(x\right)=c_{i}$ for k=0,1,…,n and some *i*. This proves:

**Proposition** **2.** *Let $f\in M_{l}\left(I\right)$ and n≥1. Then $f^{n}$ has local extrema at the critical points and their preimages up to order n−1.*

For n≥1, let

(3) $$s_{n,i}=\#\left\{x\in \left(a,b\right):f^{n}\left(x\right)=c_{i},\;\;\;f^{k}\left(x\right)\neq c_{j}\;\mathrm{for}\;0\leq k\leq n-1,\;1\leq j\leq l\right\},$$

i.e., the number of interior simple zeros of the function $f^{n}\left(x\right)-c_{i}$, and set

(4) $$s_{n}=\sum_{i=1}^{l}s_{n,i}$$

for the total number of such zeros. For the convenience of notation, definition (3) can be extended to n=0: $s_{0,i}=\#\left\{x\in \left(a,b\right):x=c_{i}\right\}=1$, so that $s_{0}=l$.

In the case of differentiable maps (to be considered in Section 3, Section 4 and Section 5), $s_{n,i}$ amounts geometrically to the number of transverse intersections of $y=f^{n}\left(x\right)$ with the *i*th critical line $y=c_{i}$. Indeed, by the chain rule of derivation,

(5) $$\frac{df^{n}}{dx}\left(x\right)=\prod_{k=0}^{n-1}\frac{df}{dx}\left(f^{k}\left(x\right)\right).$$

Therefore, if $f^{k}\left(x\right)\neq c_{j}$ for all 0≤k≤n−1 and 1≤j≤l, then $df^{n}\left(c_{i}\right)/dx\neq 0$. A solution $x^{*}$ of $f^{n}\left(x\right)-c_{i}=0$ such that $df^{n}\left(x^{*}\right)/dx=0$ corresponds to a tangential intersection of the curve $y=f^{n}\left(x\right)$ with the critical line $y=c_{i}$. Abusing the language, we will speak of transverse and non-transverse intersections in the general case too. Incidentally, Equation (5) proves Proposition 2 for differentiable maps.

Next, let $e_{n}$ be the number of local extrema of $f^{n}$.

**Proposition** **3.** *Let $f\in M_{l}\left(I\right)$ and n≥0. Then,*

(6) $$e_{n+1}=e_{n}+s_{n}.$$

**Proof.**  If n=0, then $e_{0}=0$ and $s_{0}=l$, so that $e_{0}+s_{0}$ gives the right answer $e_{1}=l$.

Suppose now that n≥1 and $f^{n+1}$ has a local extremum at $x_{0}\in I$, so that $e_{n+1}$ is the number of such $x_{0}$’s. According to Proposition 1, there are two exclusive possibilities:

(a) $f^{n}\left(x_{0}\right)=c_{i}$ for some 1≤i≤l (Proposition 1(i)); or

(b) $f^{n}\left(x_{0}\right)\neq c_{i}$ for all 1≤i≤l and $f^{n}$ has a local extremum at $x_{0}$ (Proposition 1(ii) and (iii)).

In turn, (a) subdivides according to whether $x_{0}$ is a transverse or a tangential intersection of $y=f^{n}\left(x\right)$ with the critical line $y=c_{i}$:

(a1) $f^{n}\left(x_{0}\right)=c_{i}$ and $f^{k}\left(x_{0}\right)\neq c_{j,}$ for all 0≤k≤n−1, 1≤j≤l.

(a2) $f^{n}\left(x_{0}\right)=c_{i}$ and $f^{k}\left(x_{0}\right)=c_{j}$ for some *k* and *j*, 0≤k≤n−1, 1≤j≤l.

Therefore, each $x_{0}\in I$ that contributes to $e_{n+1}$ contributes to $s_{n}$ (if case (a1) holds) or, otherwise, to $e_{n}$ (if case (a2) or (b) holds). The bottom line is Equation (6). □

Figure 1 illustrates Equation (6) for the bimodal map

(7) $$f\left(x\right)=9.375x^{3}-15.4688x^{2}+6.75x+0.1,$$

I=[0,1], whose critical points are $c_{1}=0.3$ ($f(c_{1})=0.985938$) and $c_{2}=0.8$ ($f(c_{2})=0.4$).

In the next two sections we discuss how the transverse and tangential intersections of $f^{n}$ with the critical lines are related to two salient aspects of the dynamics generated by *f*: topological entropy and superstable periodic orbits.

### 2.2. Topological Entropy

The connection of the recursive Formula (6) with the topological entropy of $f\in M_{l}\left(I\right)$, h(f), is readily established through the lap number $\ell_{n}$ of $f^{n}$, which is defined as the number of maximal monotonicity intervals of $f^{n}$. First, replace $\ell_{n}=e_{n}+1$ in (6) to obtain

(8) $$\ell_{n+1}=\ell_{n}+s_{n}.$$

The initial values $\ell_{0}=1$ and $s_{0}=l$ yield $\ell_{1}=l+1$, as it should.

Second, use the relation [18]

(9) $$h\left(f\right)=\lim_{n\to \infty }\frac{1}{n}\log\ell_{n}.$$

Since $\ell_{n}\leq {\left(l+1\right)}^{n}$ (see e.g., [9]),

(10) h(f)≤log(l+1).

Finally, Equations (8) and (9) lead then to the expression

(11) $$h\left(f\right)=\lim_{n\to \infty }\frac{1}{n}\log\left(1+\sum_{k=0}^{n-1}s_{k}\right),$$

which was first derived in [8]. For the general concept of entropy, see [19,20,21].

As a technical remark, the topological entropy of a continuous map (in particular, a multimodal map f:I→I) only depends on its non-wandering set [22]. A point x∈I is said to be non-wandering for *f* if for any neighborhood *U* of *x*, there is an integer n≥1 such that $f^{n}\left(U\right)\cap U\neq \emptyset$; otherwise, *x* is said to be a wandering point for *f*. The non-wandering set for *f* consists of all the points that are non-wandering for *f*.

Equations (9) and (11) add to other similar expressions of h(f) in terms of $e_{n}=\ell_{n}-1$, the number of *n*-periodic points, the variation of $f^{n}$ ([14], Theorem 1.1), etc. In this regard, the quantities $s_{k}$ in Equation (11) can be viewed in the following three different ways:

(**1**) Algebraically, $s_{k}$ is by definition (3)–(4) the number of interior simple zeros of the equations $f^{k}\left(x\right)-c_{i}=0$, i=1,2,…,l.

(**2**) Geometrically, $s_{k}$ is the total number of transverse intersections of the iterated map $f^{k}$ with the critical lines.

(**3**) Dynamically, $s_{k}$ is the total number of preimages of the critical points of minimal order *k*.

Whatever the interpretation, we are going to show that $s_{k}$ is a useful tool to study multimodal maps.

Several numerical algorithms for the topological entropy of multimodal maps based on Equation (11) can be found in [7,8,9], the algorithm in [9] being a variant of the algorithm in [8] and this, in turn, a simplification of the algorithm in [7]. The performance of the algorithm [8] has recently been benchmarked in [23] with favorable results. The computation of $s_{n}$ from the values of $s_{0},\ldots ,s_{n-1}$ is possible via the so-called min-max sequences [4], which are closely related to the kneading sequences [3,17]. As compared to the kneading symbols, the min-max symbols contain additional information on the minimum/maximum character of the critical values $f^{n}\left(c_{i}\right)$, 1≤i≤l, with virtually no extra computational penalty [7,8]. The geometrical properties of the min-max symbols were studied in [24] and [7] for twice-differentiable uni- and multimodal maps, respectively, and in [8,9] for just continuous multimodal maps. A brief overview is given in the Introduction of [9].

Let $f_{t}\in M_{l}\left(I_{t}\right)$ be a one-parametric family of *l*-modal maps whose parameter *t* ranges in an interval J⊂R. Denote by $s_{n}\left(t\right)$ the total number of transverse intersections of $y=f_{t}^{n}\left(t\right)$ with the critical lines. From (11) and the monotonicity of the logarithmic function it follows:

**Proposition** **4.** *Let $f_{t}\in M_{l}\left(I_{t}\right)$, and $t_{1},t_{2}\in J$ with $t_{1}<t_{2}$. Suppose $s_{n}\left(t_{1}\right)\leq s_{n}\left(t_{2}\right)$ for all $n\geq n_{0}$. Then $h\left(f_{t_{1}}\right)\leq h\left(f_{t_{2}}\right)$.*

As we will see in Section 4, Proposition 4 provides a handle to prove the monotonicity of the topological entropy for the family of quadratic maps. We mentioned already in the introduction that, according to [14,15], the existing monotonicity proofs [3,11,12,13] rely in one way or another on complex analysis. Unlike them, our approach uses real analysis. Let us remind at this point that the topological entropy of a family of unimodal maps labeled by some natural parameter (such as its critical value) is not usually monotone, even under very favorable assumptions [25]. More generally, let $f_{v}$ be a polynomial map parametrized by its critical values $v=(v_{1},\ldots ,v_{l})$. Then, according to ([14], Theorem 1.1), for l≥2 there exist fixed values of $v_{2},\ldots ,v_{l}$ such that the map $v_{1}\mapsto h\left(f_{v}\right)$ is not monotone. For multimodal maps, monotonicity of the map is replaced by the connectivity of the isentropes ([15], Theorem 1.2). See also [15] for related results and open conjectures.

### 2.3. Superstable Periodic Orbits

Let $x_{0}\in I$ and set $x_{k}=f^{k}\left(x_{0}\right)=f\left(x_{k-1}\right)$ for k≥1. Suppose for the time being that *f* is differentiable and a critical point $c_{i}$ is periodic with prime period *p*. Then, each point of the orbit $O\left(c_{i}\right)=\left\{c_{i}\equiv x_{0},x_{1},\ldots ,x_{p-1}\right\}$ is a fixed point of $f^{p}$: $f^{p}\left(x_{j}\right)=x_{j+p}=x_{j}$ for 0≤j≤p−1. $O(c_{i})$ is said to be superstable because (see Equation (5))

(12) $$\frac{df^{p}}{dx}\left(x_{j}\right)=\frac{df}{dx}\left(x_{0}\right)\frac{df}{dx}\left(x_{1}\right)\ldots \frac{df}{dx}\left(x_{p-1}\right)=0\;\;\mathrm{for}\;j=0,1,\ldots ,p-1,$$

since $df\left(x_{0}\right)/dx\equiv df\left(c_{i}\right)/dx=0$. In other words, $df^{p}\left(x_{j}\right)/dx$ (whose absolute value quantifies the stability of the fixed points $c_{i}$, $x_{1}$, …, $x_{p-1}$ of $f^{p}$ ) vanishes at each point of the periodic orbit.

On the other hand, $f^{n}$ has local extrema at all critical points for n≥1, so that the periodicity condition $f^{p}\left(c_{i}\right)=c_{i}$ amounts to a tangential intersection of the curve $y=f^{p}\left(x\right)$ and the critical line $y=c_{i}$ at $x=c_{i}$. Therefore, while the transverse intersections of $f^{n}$ with the critical lines are the only input needed to calculate the topological entropy of multimodal maps, the tangential intersections, if any, are the main ingredient of the periodic orbits (cycles) of a critical point. All in all, the intersections of $f^{n}$ with the critical lines, whether transverse or tangential, give information about the dynamical complexity and superstability of the orbits.

## 3. Application Case: Quadratic Maps

Quadratic maps have been the workhorse of chaotic dynamics for two good reasons: their dynamic exhibits a mind-boggling complexity despite being algebraically so simple and, precisely because of this simplicity, many of their dynamical properties are amenable to analytical scrutiny. We consider henceforth the family of the real quadratic maps

(13) $$q_{t}\left(x\right)=t-x^{2},$$

where x∈R and 0≤t≤2. The critical point and the critical value of $q_{t}$ are c=0 and $q_{t}\left(0\right)=t$, respectively, so the critical line y=0 is the *x*-axis in the Cartesian plane $\left\{\left(x,y\right)\in R^{2}\right\}$. The quadratic family has two fixed points,

(14) $$x_{fix,1}\left(t\right)=-\frac{1}{2}\left(1+\sqrt{1+4t}\right)\leq -1,\;\;x_{fix,2}\left(t\right)=\frac{1}{2}\left(-1+\sqrt{1+4t}\right)\geq 0.$$

Therefore, an invariant finite interval $I_{t}$, i.e., $q_{t}\left(I_{t}\right)\subset I_{t}$, where defining a dynamic generated by $q_{t}$, is

(15) $$I_{t}=\left[x_{fix,1}\left(t\right),-x_{fix,1}\left(t\right)\right]=\left[-\frac{1}{2}\left(1+\sqrt{1+4t}\right),\frac{1}{2}\left(1+\sqrt{1+4t}\right)\right].$$

It holds $I_{0}=\left[-1,1\right]\subset I_{t}\subset \left[-2,2\right]=I_{2}$. Moreover,

(16) $$q_{t}\left(-x_{fix,1}\left(t\right)\right)=q_{t}\left(x_{fix,1}\left(t\right)\right)=x_{fix,1}\left(t\right),$$

so that the boundary of $I_{t}$, $\partial I_{t}=\left\{x_{fix,1}\left(t\right),-x_{fix,1}\left(t\right)\right\}$, is also invariant: $q_{t}\left(\partial I_{t}\right)=\left\{x_{fix,1}\left(t\right)\right\}\subset \partial I_{t}$. Since all $x\notin I_{t}$ escape to −∞ under iterations of $q_{t}$, the set $I_{t}$ contains the non-wandering set of $q_{t}$.

See Figure 2 for some instances of the quadratic family. The bifurcation diagram of $q_{t}\left(x\right)$ in Figure 3 shows that the asymptotic dynamics of the quadratic family (chaotic attractors, along with stable fixed points and periodic orbits) lives in the interval −2≤x≤2. After the period-doubling cascade, chaos onset occurs at the Feigenbaum point $t_{F}=1.401155\ldots$, i.e., the topological entropy of $q_{t}$ is positive for $t>t_{F}$.

The dynamical systems generated by $q_{t}\left(x\right)$, where $x\in I_{t}$ and 0≤t≤2, and the more popular logistic maps $f_{\mu }\left(z\right)=4\mu z\left(1-z\right)$, where 0≤z≤1 and $\frac{1}{2}\leq \mu \leq 1$, are conjugate to each other via the affine transformation φ:[0,1]→[−2μ,2μ] defined as

(17) x=φ(z)=4μz−2μandt=2μ(2μ−1)

or

(18) $$z=\varphi^{-1}\left(x\right)=\frac{x}{4\mu }+\frac{1}{2}\;\;\mathrm{with}\;\mu =\frac{1}{4}\left(1+\sqrt{1+4t}\right).$$

Thus, ${\left.q_{0}\right|}_{\left[-1,1\right]}$ is conjugate to ${\left.f_{0.5}\right|}_{\left[0,1\right]}$, and ${\left.q_{2}\right|}_{\left[-2,2\right]}$ to ${\left.f_{1}\right|}_{\left[0,1\right]}$. Note that $-2\mu =x_{fix,1}$, so $I_{t}=\left[-2\mu ,2\mu \right]$.

An advantage of the quadratic map (13) is that the transverse (resp. tangential) intersections of $y=q_{t}^{n}\left(x\right)$ with the critical line correspond to the simple (resp. multiple) roots of $q_{t}^{n}\left(x\right)$, a polynomial of degree $2^{n}$. Since $q_{t}\left(x\right)$ is unimodal (l=1), Equation (4) simplifies to

(19) $$s_{n}\left(t\right)=\#\left\{x\in {\overset{˚}{I}}_{t}:q_{t}^{n}\left(x\right)=0,\;\;\;q_{t}^{k}\left(x\right)\neq 0\;\mathrm{for}\;0\leq k\leq n-1\right\},$$

where ${\overset{˚}{I}}_{t}=I_{t}\backslash \partial I_{t}$ is the interior of $I_{t}$. Therefore, $s_{n}\left(t\right)$ stands for the number of simple zeros of $q_{t}^{n}\left(x\right)$ in ${\overset{˚}{I}}_{t}$ or, equivalently, for the number of transverse intersections of the curve $y=q_{t}^{n}\left(x\right)$ with the critical line y=0. We show in Remark 1 below that ${\overset{˚}{I}}_{t}$ contains all zeros of $q_{t}^{n}\left(x\right)$, therefore ${\overset{˚}{I}}_{t}$ can be safely replaced by ${\overset{˚}{I}}_{2}=\left(-2,2\right)$ (or R, for that matter) in Equation (19).

### 3.1. Root Branches

We set out to study the real solutions of the equation $q_{t}^{n}\left(x\right)\equiv t-{\left(q_{t}^{n-1}\left(x\right)\right)}^{2}=0$, n≥1, which is a polynomial equation of degree $2^{n}$ in *x*. If $\bar{x}$ is a solution, then $-\bar{x}$ is also a solution since $q_{t}^{n}\left(-x\right)=q_{t}^{n}\left(x\right)$.

The following two cases are trivial: (i) for t=0, $q_{0}^{n}\left(x\right)\equiv -x^{2^{n}}=0$ has the $2^{n}$-fold solution x=0; (ii) for t=2, $q_{2}^{n}\left(x\right)=0$ has $2^{n}$ simple solutions in (−2,2), namely,

(20) $${\bar{x}}_{\sigma_{1},\ldots ,\sigma_{n}}=\sigma_{1}\sqrt{2+\sigma_{2}\sqrt{2+\ldots +\sigma_{n}\sqrt{2}}},$$

where $\sigma_{1},\ldots ,\sigma_{n}\in \left\{+,-\right\}$. Alternatively, the roots ${\bar{x}}_{\sigma_{1},\ldots ,\sigma_{n}}$ have the following trigonometric closed-form ([26], Problem 183):

(21) $${\bar{x}}_{\sigma_{1},\ldots ,\sigma_{n}}=2\sin\left(\frac{\pi }{4}\sum_{k=1}^{n}\frac{\sigma_{1}\sigma_{2}\cdots \sigma_{k}}{2^{k-1}}\right).$$

In the general case, consider the map $F_{n}:R\times \left[0,2\right]\to R$ defined as $F_{n}\left(x,t\right)=$$q_{t}^{n}\left(x\right)$ and the point $\left(\bar{x},\bar{t}\right)=\left({\bar{x}}_{\sigma_{1},\ldots ,\sigma_{n}},2\right)$, so that $F_{n}\left(\bar{x},\bar{t}\right)=$$q_{2}^{n}\left({\bar{x}}_{\sigma_{1},\ldots ,\sigma_{n}}\right)=0$ and

(22) $$\frac{\partial F_{n}}{\partial x}\left(\bar{x},\bar{t}\right)=\frac{dq_{\bar{t}}^{n}}{dx}\left(\bar{x}\right)=\prod_{k=0}^{n-1}\frac{dq_{2}}{dx}\left(q_{2}^{k}\left({\bar{x}}_{\sigma_{1},\ldots ,\sigma_{n}}\right)\right)\neq 0$$

since $q_{2}^{0}\left({\bar{x}}_{\sigma_{1},\ldots ,\sigma_{n}}\right)={\bar{x}}_{\sigma_{1},\ldots ,\sigma_{n}}\neq 0$ and $q_{2}^{k}\left({\bar{x}}_{\sigma_{1},\ldots ,\sigma_{n}}\right)=-{\bar{x}}_{\sigma_{k+1},\ldots ,\sigma_{n}}\neq 0$ for k=1,…,n−1. By the Implicit Function Theorem, there exists a neighborhood U⊂[0,2] of $\bar{t}=2$ and a unique smooth function $\phi_{\sigma_{1},\ldots ,\sigma_{n}}:U\to R$ such that $\phi_{\sigma_{1},\ldots ,\sigma_{n}}\left(2\right)={\bar{x}}_{\sigma_{1},\ldots ,\sigma_{n}}$ and $q_{t}^{n}\left(\phi_{\sigma_{1},\ldots ,\sigma_{n}}\left(t\right)\right)\equiv t-q_{t}^{n-1}{\left(\phi_{\sigma_{2},\ldots ,\sigma_{n}}\left(t\right)\right)}^{2}=0$, i.e.,

(23) $$\phi_{\sigma_{1},\ldots ,\sigma_{n}}\left(t\right)=\sigma_{1}\sqrt{t+\phi_{\sigma_{2},\ldots ,\sigma_{n}}\left(t\right)}=\ldots =\sigma_{1}\sqrt{t+\sigma_{2}\sqrt{t+\ldots +\sigma_{n}\sqrt{t}}},$$

for all t∈U. Therefore, in this case the “implicit” functions $\phi_{\sigma_{1},\ldots ,\sigma_{n}}\left(t\right)$ are explicitly known, and

(24) $$-2<\phi_{\sigma_{1},\ldots ,\sigma_{n}}\left(t\right)<2$$

for all n≥1, $\sigma_{1},\ldots ,\sigma_{n}\in \left\{+,-\right\}$, and 0≤t≤2.

The functions $\phi_{\sigma_{1},\ldots ,\sigma_{n}}\left(t\right)$ will be generically called root branches of $q_{t}^{n}\left(x\right)$; notice that the sign of $\phi_{\sigma_{1},\ldots ,\sigma_{n}}\left(t\right)$ depends on $\sigma_{1}$, hence $\phi_{-\sigma_{1},\sigma_{2},\ldots ,\sigma_{n}}\left(t\right)=-\phi_{\sigma_{1},\sigma_{2},\ldots ,\sigma_{n}}\left(t\right)$. When the components are not important, we shorten the notation and write $(\sigma_{1},\ldots ,\sigma_{n})=\sigma$. We call *n*, the number of components of σ, the rank of the signature σ and denote it by $\left|\sigma \right|$. Likewise, we call $\left|\sigma \right|$ the rank of $\phi_{\sigma }\left(t\right)$, so $q_{t}^{\left|\sigma \right|}\left(\phi_{\sigma }\left(t\right)\right)=0$. Sometimes we write ± in a component of a signature to refer to both branches. If σ is a final segment of the signature ρ, we say that $\phi_{\rho }\left(t\right)$ is a successor of $\phi_{\sigma }\left(t\right)$; likewise if σ is an initial segment of the signature ρ, we say the $\phi_{\rho }\left(t\right)$ is a predecessor of $\phi_{\sigma }\left(t\right)$.

Let us pause at this point to address a few basic properties of the root branches. We denote by $\mathrm{dom}\;\phi_{\sigma }$ the definition domain of $\phi_{\sigma }\left(t\right)$, that is, the points in the parametric interval [0,2] where the right hand side of Equation (23) exists. In view of (23), the definition domains of $\phi_{\pm ,\sigma }\left(t\right)$, the two immediate successors of $\phi_{\sigma }\left(t\right)$, are given by

(25) $$\mathrm{dom}\;\phi_{+,\sigma }=\mathrm{dom}\;\phi_{-,\sigma }=\left\{0\leq t\leq 2:t+\phi_{\sigma }\left(t\right)\geq 0\right\}.$$

Since $\phi_{\sigma }\left(0\right)=0$ for all signatures σ and $\phi_{\sigma }\left(2\right)={\bar{x}}_{\sigma_{1},\ldots ,\sigma_{n}}\in \left(-2,2\right)$, it holds $\left\{0,2\right\}\subset \mathrm{dom}\;\phi_{\pm ,\sigma }$ for all root branches. It is also obvious that

(26) $$\mathrm{dom}\;\phi_{+,\sigma }=\mathrm{dom}\;\phi_{-,\sigma }\subset \mathrm{dom}\;\phi_{\sigma },$$

so that the consecutive successors of $\phi_{\sigma }\left(t\right)$ have, in general, ever smaller definition domains. The only exceptions are

(27) $$\mathrm{dom}\;\phi_{+,+,\ldots ,+}=\mathrm{dom}\;\phi_{-,+,\ldots ,+}=\left[0,2\right].$$

Examples of definition domains are the following:

(28) $$\begin{array}{cc} \mathrm{dom}\;\phi_{+,-} & =\{0\}\cup [1,2],\\ \mathrm{dom}\;\phi_{+,-,+} & =\{0\}\cup [1.7549\ldots ,2],\\ \mathrm{dom}\;\phi_{+,-,+,-} & =\{0\}\cup \{1\}\cup [1.3107\ldots ,2]. \end{array}$$

Figure 4 shows the graphs of the root branches of ranks 1 to 5. In panel (a), the 2-fold zero $\phi_{\pm }\left(0\right)=0$ correspond to the tangential intersection of $q_{0}\left(x\right)$ with the *x*-axis in Figure 2a, while the 2-fold zero $\phi_{\pm -}\left(1\right)=0$ and the two simple zeros $\phi_{++}\left(1\right)=\sqrt{2}$ and $\phi_{-+}\left(1\right)=-\sqrt{2}$ correspond to the tangential intersection and the two transverse intersections, respectively, of $q_{1}^{2}\left(x\right)$ with the *x*-axis in Figure 2b. In panel (b), the two simple roots $\phi_{+++}\left(1\right)=1.5538$ and $\phi_{-++}\left(1\right)=-1.5538$ correspond to the transverse intersections of $q_{1}^{3}\left(x\right)$ with the *x*-axis in Figure 2c, while the two 2-fold roots $\phi_{+\pm -}\left(1\right)=1$ and $\phi_{-\pm -}\left(1\right)=-1$ correspond to the two tangential intersections of $q_{1}^{3}\left(x\right)$. The 16 roots $\phi_{\sigma_{1},\sigma_{2},\sigma_{3},\sigma_{4}}\left(2\right)$ in panel (c) correspond to the 16 transverse intersections of $q_{2}^{4}\left(x\right)$ with the *x*-axis in Figure 2d. Finally, panel (d) shows together the 62 root branches of ranks 1 to 5.

In the panels of Figure 4 we see that the $2^{\left|\sigma \right|}$ root branches $\phi_{\sigma }\left(t\right)$, $1\leq \left|\sigma \right|\leq 5$, build $2^{\left|\sigma \right|-1}$ parabola-like curves, which we denote $\phi_{\sigma_{1},\ldots ,\sigma_{i-1},\pm ,\sigma_{i+1},\ldots ,\sigma_{n}}\left(t\right)$ (1≤i≤n), this notation meaning that the curves $\phi_{\sigma_{1},\ldots ,\sigma_{i}=+,\ldots ,\sigma_{n}}\left(t\right)$ and $\phi_{\sigma_{1},\ldots ,\sigma_{i}=-,\ldots ,\sigma_{n}}\left(t\right)$ (the branches of the parabola) emerge from a common vertex $\left(t_{b},\phi_{\sigma_{1},\ldots ,\sigma_{i}=+,\ldots ,\sigma_{n}}\left(t_{b}\right)\right)=\left(t_{b},\phi_{\sigma_{1},\ldots ,\sigma_{i}=-,\ldots ,\sigma_{n}}\left(t_{b}\right)\right)$ with a vertical tangent. The vertex and the abscissa $t_{b}$ will be called indistinctly branching point (geometrical terminology) or bifurcation point (dynamical terminology) of the parabola or any of its branches. Root parabolas with the vertex on the *t*-axis, $\phi_{\pm ,\sigma_{2},\ldots ,\sigma_{n}}\left(t\right)$, are sometimes called on-line parabolas, otherwise off-axis parabolas. The branching point $t_{b}$ has also a direct geometrical interpretation in state space: the curve $q_{t_{b}}^{\left|\sigma \right|}\left(x\right)$ intersects tangentially the *x*-axis (the critical line) at the point $x=\phi_{\sigma_{1},\ldots ,\sigma_{i-1},\pm ,\sigma_{i+1},\ldots ,\sigma_{n}}\left(t_{b}\right)$. The opening of the branches to the right means that, if the contact occurs from the upper half-plane as *t* increases, the corresponding local extremum is a minimum, whereas if the contact occurs from the lower half-plane, it is a maximum. In panel (d) of Figure 4 we see that different branches do not cross but touch at the bifurcation points (“T-crossings”). We will show below that all these properties hold in general.

### 3.2. Smoothness Domains of the Root Branches

A crucial issue for our purposes is the distinction between $\mathrm{dom}\;\phi_{\sigma }$ and $\mathrm{sdom}\;\phi_{\sigma }$, the subset of $\mathrm{dom}\;\phi_{\sigma }$ where $\phi_{\sigma }\left(t\right)$ is smooth. As it will turn out in Section 4, $\mathrm{sdom}\;\phi_{\sigma }$ comprises the parametric values *t* for which the root $\phi_{\sigma }\left(t\right)$ is simple—precisely the *t*’s that count for $s_{\left|\sigma \right|}\left(t\right)$, Equation (19). Therefore $\mathrm{sdom}\;\phi_{\sigma }$ can be read not only as “smoothness domain” but also as “simplicity domain”.

To learn about $\mathrm{sdom}\;\phi_{\sigma }$, we go back to the neighborhood U⊂[0,2] of t=2 where the $2^{n}$ distinct root branches $\phi_{\sigma_{1},\ldots ,\sigma_{n}}\left(t\right)$ are locally defined and continuously differentiable. This neighborhood can be extended to include lower and lower *t* values as long as $\partial q_{t}^{n}\left(\phi_{\sigma_{1},\ldots ,\sigma_{n}}\left(t\right)\right)/\partial x\neq 0$, i.e., as long as $\phi_{\sigma_{1},\ldots ,\sigma_{n}}\left(t\right)$ has not a vertical tangent. Since

(29) $$\frac{\partial q_{t}^{n}}{\partial x}\left(x\right)=\prod_{k=0}^{n-1}\frac{dq_{t}}{dx}\left(q_{t}^{k}\left(x\right)\right)={\left(-2\right)}^{n}xq_{t}\left(x\right)\cdots q_{t}^{n-1}\left(x\right),$$

the obstruction $\partial q_{t}^{n}\left(\phi_{\sigma_{1},\ldots ,\sigma_{n}}\left(t\right)\right)/\partial x=0$ occurs whichever condition

**(C)**$q_{t}^{k}\left(\phi_{\sigma_{1},\ldots ,\sigma_{n}}\left(t\right)\right)=0$ (0≤k≤n−1)

is fulfilled first. Conditions (**C**) comprise those parametric values *t* for which $\phi_{\sigma_{1},\ldots ,\sigma_{n}}\left(t\right)$ is the critical point (k=0) and, for n≥2, any of its, at most $2^{n}-2$, preimages up to order n−1.

If k=0, then $q_{t}^{0}\left(\phi_{\sigma_{1},\ldots ,\sigma_{n}}\left(t\right)\right)\equiv \phi_{\sigma_{1},\ldots ,\sigma_{n}}\left(t\right)=0$. If 1≤k≤n−1 (n≥2), then use Equation (23) to derive

(30) $$q_{t}\left(\phi_{\sigma_{1},\ldots ,\sigma_{n}}\left(t\right)\right)=t-\phi_{\sigma_{1},\ldots ,\sigma_{n}}{\left(t\right)}^{2}=-\phi_{\sigma_{2},\ldots ,\sigma_{n}}\left(t\right)$$

and, in general,

(31) $$q_{t}^{k}\left(\phi_{\sigma_{1},\ldots ,\sigma_{n}}\left(t\right)\right)=q_{t}\left(-\phi_{\sigma_{k},\ldots ,\sigma_{n}}\left(t\right)\right)=-\phi_{\sigma_{k+1},\ldots ,\sigma_{n}}\left(t\right),$$

so the conditions (**C**) amount to:

**(C’)**$\phi_{\sigma_{k+1},\ldots ,\sigma_{n}}\left(t\right)=0$ (0≤k≤n−1).

Note that

(32) $$\phi_{\sigma_{k+1},\ldots ,\sigma_{n}}\left(t_{b}\right)=0\;\Rightarrow \;\phi_{-\sigma_{k+1},\sigma_{k+2}\ldots ,\sigma_{n}}\left(t_{b}\right)=-\phi_{\sigma_{k+1},\sigma_{k+2}\ldots ,\sigma_{n}}\left(t_{b}\right)=0,$$

therefore,

(33) $$\phi_{\sigma_{k+1},\ldots ,\sigma_{n}}\left(t_{b}\right)=0\;\Rightarrow \;\left\{\begin{array}{cc} \phi_{\pm \sigma_{1},\ldots ,\sigma_{n}}\left(t_{b}\right)=0 & \mathrm{if}\;k=0,\\ \phi_{\sigma_{1},\ldots ,\pm \sigma_{k+1},\ldots ,\sigma_{n}}\left(t_{b}\right)=\phi_{\sigma_{1},\ldots ,\sigma_{k}}\left(t_{b}\right) & \mathrm{if}\;1\leq k\leq n-1, \end{array}\right.$$

which means that 0 (k=0) or $\phi_{\sigma_{1},\ldots ,\sigma_{k}}\left(t_{b}\right)$ (1≤k≤n−1) is a multiple zero of $q_{t_{b}}^{n}\left(x\right)$. Such a point $t_{b}$ is a branching (or bifurcation) point of $\phi_{\sigma_{1},\ldots ,\pm \sigma_{k+1},\ldots ,\sigma_{n}}\left(t\right)$ if both branches are defined in a neighborhood of $t_{b}$; otherwise, $t_{b}$ is an isolated point of $\mathrm{dom}\;\phi_{\sigma_{1},\ldots ,\pm \sigma_{k+1},\ldots ,\sigma_{n}}$ (actually, one can check that the isolated points of $\phi_{\sigma }\left(t\right)$, if any, correspond to branching points of some predecessor). Branching points and isolated points are called singular points; the complement are the regular points of the corresponding root parabola or branches. This proves the following result:

**Proposition** **5.** *The singular points $t_{b}$ of $\mathrm{dom}\;\phi_{\sigma }$ correspond to multiple zeros of $q_{t_{b}}^{n}\left(x\right)$. In either case, $\phi_{\sigma_{k+1},\ldots ,\sigma_{n}}\left(t_{b}\right)=0$ for some k=0,1,…,n−1.*

Furthermore, if $\left|\sigma \right|=s\geq r=\left|\rho \right|$ and $\phi_{\sigma }\left(t_{0}\right)=\phi_{\rho }\left(t_{0}\right)$, i.e.,

(34) $$\sigma_{1}\sqrt{t_{0}+\phi_{\sigma_{2},\ldots ,\sigma_{s}}\left(t_{0}\right)}=\rho_{1}\sqrt{t_{0}+\phi_{\rho_{2},\ldots ,\rho_{r}}\left(t_{0}\right)},$$

then

(35) $$\phi_{\sigma }\left(t_{0}\right)=\phi_{\rho }\left(t_{0}\right)=0\;\;\mathrm{if}\;\sigma_{1}\neq \rho_{1};$$

otherwise, keep squaring the Equation (34) and recursively applying Equation (35) to the resulting equalities to derive:

(36) $$\sigma_{i}\neq \rho_{i}\;\mathrm{for}\;\mathrm{some}\;1\leq i\leq r\;\;\Rightarrow \;\;\phi_{\sigma_{i},\ldots ,\sigma_{s}}\left(t_{0}\right)=\phi_{\rho_{i},\ldots ,\rho_{r}}\left(t_{0}\right)=0,$$

or else

(37) $$\sigma_{i}=\rho_{i}\;\mathrm{for}\;1\leq i\leq r\;\mathrm{and}\;s>r\;\;\Rightarrow \;\;\phi_{\sigma_{r+1},\ldots ,\sigma_{s}}\left(t_{0}\right)=0.$$

By Proposition 5, $t_{0}$ is a singular point of $\phi_{\sigma }\left(t\right)$. We conclude:

**Proposition** **6.** *A root branch $\phi_{\sigma }\left(t\right)$ can be smoothly extended from the boundary t=2 to a maximal interval $\mathrm{sdom}\;\phi_{\sigma }:=\left(t_{\sigma },2\right]$, where $t_{\sigma }=\max\{t\in \left[0,2\right):\phi_{\sigma_{k+1},\ldots ,\sigma_{n}}\left(t\right)=0$ for some 0≤k≤n−1} is a branching point of $\phi_{\sigma }\left(t\right)$. Moreover, $\phi_{\sigma }\left(t\right)\neq \phi_{\rho }\left(t\right)$ for σ≠ρ and t∈$\mathrm{sdom}\;\phi_{\sigma }\cap \mathrm{sdom}\;\phi_{\rho }$.*

In other words, root branches do not cross or touch in their smoothness domains. Table 1, obtained from Figure 4, lists the smoothness domains $\left(t_{\sigma },2\right]$ of the 15 root parabolas up to rank 4.

The ordering of the branching points $t_{\sigma }$ is related to the ordering of the root branches. Due to the strictly increasing/decreasing monotonicity of the positive/negative square root function, $\phi_{\sigma }\left(t\right)<\phi_{\rho }\left(t\right)$ implies

(38) $$\phi_{-,\rho }\left(t\right)<\phi_{-,\sigma }\left(t\right)<\phi_{+,\sigma }\left(t\right)<\phi_{+,\rho }\left(t\right).$$

Thus, attaching a sign “+” (resp. “−”) in front the signature preserves (resp. reverses) the ordering. This generalizes to the following signed lexicographical order for root branches.

**Proposition** **7.** *Given σ≠ρ with $\left|\sigma \right|\geq \left|\rho \right|$ and t∈$\mathrm{sdom}\;\phi_{\sigma }\cap \mathrm{sdom}\;\phi_{\rho }$, the following holds.*

*(a) If $\sigma_{1}\neq \rho_{1}$ then*

(39) $$\phi_{\sigma }\left(t\right)\left\{\begin{array}{cc} >\phi_{\rho }\left(t\right) & \mathrm{if}\;\sigma_{1}=+1,\\ <\phi_{\rho }\left(t\right) & \mathrm{if}\;\sigma_{1}=-1. \end{array}\right.$$

*(b) If $\sigma_{i}=\rho_{i}$ for i=1,…,k, and $k=\left|\rho \right|$ or $\sigma_{k+1}\neq \rho_{k+1}$, then*

(40) $$\phi_{\sigma }\left(t\right)\left\{\begin{array}{cc} >\phi_{\rho }\left(t\right) & \mathrm{if}\;\sigma_{1}\times \ldots \times \sigma_{k+1}=+1,\\ <\phi_{\rho }\left(t\right) & \mathrm{if}\;\sigma_{1}\times \ldots \times \sigma_{k+1}=-1. \end{array}\right.$$

Since root branches do not cross or touch in their smoothness domains, they can be ordered alternatively by $\phi_{\sigma }\left(2\right)$. According to Equation (33), the inequalities (39) and (40) can turn equalities at a common singular point of $\mathrm{dom}\;\phi_{\sigma }\cap \mathrm{dom}\;\phi_{\rho }$.

As an example,

(41) $$\phi_{-,{\left\{+\right\}}^{n-1}}\left(t\right)<\phi_{-,\sigma }\left(t\right)<\phi_{-,-,{\left\{+\right\}}^{n-2}}\left(t\right)<\phi_{+,-,{\left\{+\right\}}^{n-2}}\left(t\right)<\phi_{+,\sigma }\left(t\right)<\phi_{{\left\{+\right\}}^{n}}\left(t\right),$$

for all $t\in \mathrm{sdom}\;\phi_{\pm ,\sigma }$, where $\left|\sigma \right|=n-1$. Equation (41) shows the upper and lower bounds of the positive and negative root branches.

**Remark** **1.** *According to Equation (41),*

(42) $$\lim_{n\to \infty }\phi_{{\left\{+\right\}}^{n}}\left(t\right)=\frac{1}{2}\left(1+\sqrt{1+4t}\right)=:\phi_{{\left\{+\right\}}^{\infty }}\left(t\right)$$

*and*

(43) $$\lim_{n\to \infty }\phi_{-,{\left\{+\right\}}^{n-1}}\left(t\right)=-\lim_{n\to \infty }\phi_{{\left\{+\right\}}^{n-1}}\left(t\right)=-\frac{1}{2}\left(1+\sqrt{1+4t}\right)=:\phi_{-,{\left\{+\right\}}^{\infty }}\left(t\right)$$

*are the optimal upper and lower bounds, respectively, of all root branches for t>0. From Equations (15) and (18) we see that*

(44) $$\left[\phi_{-,{\left\{+\right\}}^{\infty }}\left(t\right),\phi_{{\left\{+\right\}}^{\infty }}\left(t\right)\right]=I_{t}=\left[-2\mu ,2\mu \right],$$

*where μ=$\frac{1}{4}\left(1+\sqrt{1+4t}\right)$ is the parameter value of the logistic map ${\left.f_{\mu }\right|}_{\left[0,1\right]}$ conjugate to ${\left.q_{t}\right|}_{I_{t}}$. Therefore, all zeros of $q_{t}^{n}\left(x\right)$ are in the open interval ${\overset{˚}{I}}_{t}=\left(-2\mu ,2\mu \right)=\left(-\frac{1}{2}\left(1+\sqrt{1+4t}\right),\frac{1}{2}\left(1+\sqrt{1+4t}\right)\right)$ for n≥1.*

## 4. Application I: Monotonicity of the Topological Entropy

In Section 3, the smooth root branch $\phi_{\sigma }\left(t\right)$ was extended from a neighborhood of the boundary t=2 to a maximal interval $\mathrm{sdom}\;\phi_{\sigma }=\left(t_{\sigma },2\right]$, called the smoothness domain of $\phi_{\sigma }$. The next Proposition excludes the possibility that $\phi_{\sigma }\left(t\right)$ is also defined in an interval $\left(t_{1},t_{2}\right)$ with $0\leq t_{1}<t_{2}\leq t_{\sigma }$. By the same arguments used with $\mathrm{sdom}\;\phi_{\sigma }$, the endpoints $t_{1}$ and $t_{2}$ would be then branching points of $\phi_{\sigma }\left(t\right)$.

**Proposition** **8.** *For all $\left|\sigma \right|\geq 1$, $\mathrm{dom}\;\phi_{\sigma }$ does not include intervals other than $[t_{\sigma },2].$*

**Proof.**  Suppose that the, say positive, root branch $\phi_{\sigma_{1},\ldots ,\sigma_{n}}\left(t\right)$ is also defined in an interval $\left(t_{1},t_{2}\right)\subset \left[0,2\right]$, where $0\leq t_{1},t_{2}\leq t_{\sigma }$ are two branching points, hence, $\phi_{\sigma_{k_{1}+1},\ldots ,\sigma_{n}}\left(t_{1}\right)=\phi_{\sigma_{k_{2}+1},\ldots ,\sigma_{n}}\left(t_{2}\right)=0$ for some $0\leq k_{1},k_{2}\leq n-1$ (Proposition 6). In this case, the positive root branches $\phi_{\sigma_{1},\ldots ,\pm \sigma_{k_{1}+1},\ldots ,\sigma_{n}}\left(t\right)$ and $\phi_{\sigma_{1},\ldots ,\pm \sigma_{k_{2}+1},\ldots ,\sigma_{n}}\left(t\right)$ would compose the two parabolas depicted in Figure 5a in a neighborhood of $t_{1}$ and $t_{2}$, respectively.

The “⊂” bifurcation at “time” $t_{1}$ corresponds to a local minimum (resp. local maximum) of $q_{t}^{n}\left(x\right)$ crossing the *x*-axis from above (resp. below) at the point $x_{1}=\phi_{\sigma_{1},\ldots ,\sigma_{n}}\left(t_{1}\right)=0$ if $k_{1}=0$ or $x_{1}=\phi_{\sigma_{1},\ldots ,\sigma_{k_{1}}}\left(t_{1}\right)>0$ if $k_{1}\geq 1$ (see Figure 5b,c and Equation (33)). Bifurcation points with branches opening to the right occur at the left endpoint of the smoothness domains, in particular at t=0, so they are certainly allowed.

The “⊃” bifurcation at “time” $t_{2}$ corresponds to local a minimum (resp. maximum) of $q_{t}^{n}\left(x\right)$ crossing the *x*-axis from below (resp. above) at the point $x_{2}=\phi_{\sigma_{1},\ldots ,\sigma_{n}}\left(t_{2}\right)=0$ if $k_{2}=0$ or $x_{2}=\phi_{\sigma_{1},\ldots ,\sigma_{k_{2}}}\left(t_{2}\right)>0$ (see Figure 5b,c and Equation (33)). To show that bifurcation points with branches opening to the left, however, are not allowed, we are going to exploit the following *Fact* derived from the hypothetical existence of ⊃ bifurcations.

*Fact*: In both cases illustrated in Figure 5b (where $q_{t_{2}}^{n}\left(x_{2}\right)$ is a local minimum and ${\left.\partial q_{t}^{n}\left(x_{2}\right)/\partial t\right|}_{t=t_{2}}\geq 0$) and Figure 5c (where $q_{t_{2}}^{n}\left(x_{2}\right)$ is a local maximum ${\left.\partial q_{t}^{n}\left(x_{2}\right)/\partial t\right|}_{t=t_{2}}\leq 0$), given any neighborhood of $x_{2}$, $\left(x_{2}-\varepsilon ,\;x_{2}+\varepsilon \right)$ with ε>0, there exists τ>0 such that $q_{t_{2}-△t}^{n}\left(x\right)$ changes sign in $\left(x_{2}-\varepsilon ,\;x_{2}+\varepsilon \right)$ for all 0<△t≤τ because, by assumption, $q_{t}^{n}\left(x\right)$ intersects transversally the *x*-axis just before $t=t_{2}.$

It is even more true: said change of sign occurs both in $\left(x_{2}-\varepsilon ,\;x_{2}\right)$ due to the left branch, and in $\left(x_{2},\;x_{2}+\varepsilon \right)$ due to the right branch. Note that the above *Fact* does not hold for ⊂ bifurcations.

Therefore, we will consider only the first case (Figure 5b with $x=x_{2}$ and $t=t_{2}$). There are several subcases.

(a) If ${\left.\partial q_{t}^{n}\left(x_{2}\right)/\partial t\right|}_{t=t_{2}}>0$, then

(45) $$q_{t_{2}-△t}^{n}\left(x_{2}\pm △x\right)=-\partial_{t}q_{t_{2}}^{n}\left(x_{2}\right)\cdot △t+O\left(2\right),$$

where 0<△x≤ε, 0<△t≤τ, and we wrote $\partial_{t}q_{t_{2}}^{n}\left(x_{2}\right)$$\equiv {\left.\partial_{t}q_{t}^{n}\left(x_{2}\right)\right|}_{t=t_{2}}$ for brevity. From Equation (45) it follows $q_{t_{i}-△t}^{n}\left(x\right)<0$ in $\left(x_{2}-\varepsilon ,\;x_{2}+\varepsilon \right)$ for all △t, which contradicts the above *Fact*. This excludes the possibility of having a ⊃ bifurcation at $t_{2}$ if the “velocity” of $q_{t}^{n}\left(x_{2}\right)$ at $t=t_{2}$ is positive.

(b) Suppose now $\partial q_{t_{2}}^{n}\left(x_{2}\right)/\partial t=0$, so

(46) $$q_{t_{2}-\Delta t}^{n}\left(x_{2}\pm △x\right)=\frac{1}{2}\partial_{xx}q_{t_{2}}^{n}\left(x_{2}\right){\left(△x\right)}^{2}\mp \partial_{xt}^{2}q_{t_{2}}^{n}\left(x_{2}\right)△x△t+\frac{1}{2}\partial_{tt}q_{t_{2}}^{n}\left(x_{2}\right){\left(△t\right)}^{2}+O\left(3\right),$$

where $\partial_{xx}q_{t_{2}}^{n}\left(x_{2}\right)\equiv {\left.\partial_{xx}q_{t_{2}}^{n}\left(x\right)\right|}_{x=x_{2}}\geq 0$ (because $q_{t_{2}}^{n}\left(x_{2}\right)$ is a minimum in the case we are considering), $\partial_{tt}q_{t_{2}}^{n}\left(x_{2}\right)\equiv {\left.\partial_{tt}q_{t}^{n}\left(x_{2}\right)\right|}_{t=t_{2}}$ and similarly for the mixed term.

(b1) If $\partial_{xx}q_{t_{2}}^{n}\left(x_{2}\right)>0$ and some of the other O(2) terms is not zero, let △t→0 while △x is fixed to conclude from Equation (46) that $q_{t_{2}-\Delta t}^{n}\left(x_{2}\pm △x\right)$ does not change sign for sufficiently small △t, 0<△t≪△x, in contradiction to the above Fact. The same contraction follows, of course, if all O(2) terms in Equation (46) except $\partial_{xx}q_{t_{2}}^{n}\left(x_{2}\right)$ vanish.

(b2) If all terms O(2) vanish at $x=x_{2}$ and $t=t_{2}$, repeat the same argument with the terms O(3). Since $q_{t_{2}}^{n}\left(x_{2}\right)$ is a minimum and $q_{t}^{n}\left(x\right)$ is a polynomial of degree $2^{n}$, it holds $\partial_{x^{m}}^{m}q_{t_{2}}^{n}\left(x_{2}\right)>0$ for some $2\leq m\leq 2^{n}$. □

A conclusion of the proof of Proposition 8 is that the root branches do not have bifurcations with branches that open to the left or bifurcations that open to the right except for the one at the left endpoint of the smoothness domain. As a result:

**Proposition** **9.** *For all $\left|\sigma \right|\geq 1$, $\mathrm{sdom}\;\phi_{\sigma }=\left(t_{\sigma },2\right]$, where $0\leq t_{\sigma }<2$ is the unique branching point of $\phi_{\sigma }$.*

**Remark** **2.** *The images of the critical point build a sequence of polynomials $P_{n}\left(t\right):=q_{t}^{n}\left(0\right)$, that is, $P_{n}\left(t\right)$ is the nth image of 0 under $q_{t}$. Alternatively, one can define polynomial maps $P_{n}:\left[0,2\right]\to \left[-2,2\right]$ by the recursion*

(47) $$P_{0}\left(t\right)=0,\;\;P_{n}\left(t\right)=t-P_{n-1}{\left(t\right)}^{2}\;\;\mathrm{for}\;n\geq 1.$$

*Therefore, $P_{n}\left(t\right)$ is a polynomial of degree $2^{n-1}$ for n≥1, and*

(48) $$P_{n+k}\left(t\right)=q_{t}^{n+k}\left(0\right)=q_{t}^{n}\left(q_{t}^{k}\left(0\right)\right)=q_{t}^{n}\left(P_{k}\left(t\right)\right).$$

*The first polynomials are:*

(49) $$\begin{array}{c} P_{1}\left(t\right)=t\\ P_{2}\left(t\right)=t-t^{2}\\ P_{3}\left(t\right)=t-t^{2}+2t^{3}-t^{4}\\ P_{4}\left(t\right)=t-t^{2}+2t^{3}-5t^{4}+6t^{5}-6t^{6}+4t^{7}-t^{8} \end{array}$$

*If, as in the proof of Proposition 8, we interpret the parameter t as time, then the time of passage of $q_{t}^{n}\left(0\right)$ through the x-axis is given by the zeros of $P_{n}\left(t\right)=0$. Note that*

(50) $$P_{n}\left(0\right)=0\;\;\mathrm{for}\;n\geq 1,$$

*while*

(51) $$P_{1}\left(2\right)=2,\;P_{n}\left(2\right)=-2\;\;\mathrm{for}\;n\geq 2$$

*(see Figure 2d for n=4). In physical terms, $q_{t}\left(0\right)$ moves upwards from x=0 (t=0) to x=2 (t=2) at constant speed ${\dot{P}}_{1}\left(t\right)=1$ (the dot denotes time derivative), while, for n≥2, $q_{t}^{n}\left(0\right)$ moves from x=0 (t=0) to x=−2 (t=2), reversing the speed when ${\dot{P}}_{n}\left(t\right)=0$ and crossing the x-axis when $P_{n}\left(t\right)=0$. In Section 5 we will come back to these polynomials from a different perspective.*

At this point we have already cleared our way to the monotonicity of the topological entropy for the quadratic family,

(52) $$h\left(q_{t}\right)=\lim_{n\to \infty }\frac{1}{n}\log\left(1+\sum_{k=0}^{n-1}s_{k}\left(t\right)\right),$$

where $s_{k}\left(t\right)$ is the number of simple zeros of $q_{t}^{k}\left(x\right)$ or, equivalently, the number of transverse intersections of the curve $y=q_{t}^{k}\left(x\right)$ with the critical line y=0 (the *x*-axis); see Equation (19). According to Equation (41) and Remark 1, all zeros of $q_{t}^{k}\left(x\right)$ are in the interval $\left[\phi_{-,{\left\{+\right\}}^{k-1}}\left(t\right),\phi_{{\left\{+\right\}}^{k}}\left(t\right)\right]$$\subset \left(\phi_{-,{\left\{+\right\}}^{\infty }}\left(t\right),\phi_{{\left\{+\right\}}^{\infty }}\left(t\right)\right)={\overset{˚}{I}}_{t}$.

It follows from Propositions 5 and 9, that, for each signature σ with $\left|\sigma \right|=n$, $\mathrm{dom}\;\phi_{\sigma }\backslash \mathrm{sdom}\;\phi_{\sigma }$ comprises multiple roots of $q_{t}^{n}\left(t\right)$ (isolated points and the branching point $t_{\sigma }$), while the roots $\phi_{\sigma }\left(t\right)$ are simple for $t_{\sigma }<t\leq 2$ by Proposition 6. The bottom line is:

**Proposition** **10.** *The smoothness domain $\mathrm{sdom}\;\phi_{\sigma }$ comprises the values of t for which the root $\phi_{\sigma }\left(t\right)$ of $q_{t}^{\left|\sigma \right|}\left(x\right)$ is simple.*

For this reason we anticipated at the beginning of Section 3.2 that $\mathrm{sdom}\;\phi_{\sigma }$ may be called the simplicity domain of $\phi_{\sigma }$ as well. This being the case, each root $\phi_{\sigma }\left(t\right)$ contributes 0 or 1 to $s_{n}\left(t\right)$, the number of simple zeros of $q_{t}^{n}\left(x\right)$, depending on whether $0<t\leq t_{\sigma }$ or $t_{\sigma }<t\leq 2$, respectively. We conclude that

(53) $$s_{n}\left(t\right)=\sum_{\sigma \in {\left\{+,-\right\}}^{n}}\chi_{\left(t_{\sigma },2\right]}\left(t\right)=2\sum_{\rho \in {\left\{+,-\right\}}^{n-1}}\chi_{\left(t_{+,\rho },2\right]}\left(t\right)$$

for n≥1, where we used $t_{-\sigma_{1},\sigma_{2},\ldots ,\sigma_{n}}=t_{\sigma_{1},\sigma_{2},\ldots ,\sigma_{n}}$, and $\chi_{\left(t_{\sigma },2\right]}\left(t\right)$ is the characteristic or indicator function of the interval $\left(t_{\sigma },2\right]$ (1 if $t\in (t_{\sigma },2]$, 0 otherwise). Equation (53) proves:

**Theorem** **1.** *The function $s_{n}:\left[0,2\right]$$\to \{0,2,4,\ldots ,2^{n}\}$, defined in Equation (19), is piecewise constant and monotonically increasing for every n≥1. Its discontinuities occur at the branching points $t_{\sigma }$ of the root branches $\phi_{\sigma }\left(t\right)$ with $\left|\sigma \right|=n$, where $s_{n}\left(t\right)$ is lower semicontinuous.*

Figure 6 shows the function $s_{4}\left(t\right)$ based on Figure 4c. Apply now Proposition 4 to prove Milnor’s Monotonicity Conjecture for the quadratic family:

**Theorem** **2.** *The topological entropy of $q_{t}$ is a monotonically increasing function of t.*

Figure 7 shows the topological entropy of $q_{t}$ superimposed on the bifurcation diagram (Figure 3); in particular, $h(q_{t})>0$ (i.e., $q_{t}$ is chaotic) for $t>t_{F}=1.4011551\ldots$ (Feigenbaum point) and $h(q_{2})=\log2$, the highest value that the topological entropy of a unimodal map can take, see Equation (10). It can be proved that the function $t\mapsto h(q_{t})$ is a Devil’s staircase, meaning that it is continuous, monotonically increasing (Theorem 2), but there is no interval of parameters where it is strictly increasing [27,28]. The plateaus where $h(q_{t})$ is constant correspond to intervals containing a periodic attractor and the subsequent period doubling cascade (e.g., the period-3 window, clearly visible in Figure 7). This shows that periodic orbits do not disappear as *t* increases, however, the new periodic orbits that are created do not necessarily increase $h(q_{t})$. The topological entropy in Figure 7 was computed using the general algorithm presented in [9], but see [24] for a simpler and quicker algorithm adapted to unimodal maps. The small but positive values of $h(q_{t})$ to the left of $t_{F}$ are due to the slow convergence of the algorithm.

## 5. Application II: Superstable Period Orbits

In Section 4 we studied the solutions of the equation $q_{t}^{n}\left(x\right)=0$, where the parameter *t* was thought to be fixed. In other words, we were interested in the zeros of a polynomial function of the variable *x* and, more particularly, in the values of *t* for which those zeros were simple. If we fix *x* instead, then the solutions of $q_{t}^{n}\left(x\right)=0$ are the parametric values *t* such that *x* is an *n*-order preimage of 0, which is the critical point of $q_{t}$. If, moreover, x=0, then the solutions are the parametric values *t* for which the critical value is periodic with period *n*. As explained at the beginning of Section 2.3, these orbits are called superstable because then the derivative of $q_{t}^{n}$ at each point of the periodic orbit is 0 (see Equation (12)). For the quadratic maps, $q_{t}\left(0\right)=t$, so 0 is not a fixed point for t>0.

**Remark** **3.** *If in Section 4 our main concern were the transverse intersections of $q_{t}^{n}\left(x\right)$ with the x-axis, in this section it will be the transverse intersection of the bisector with the positive root branches (if any). In this regard, note that the bisector can intersect a positive root branch $\phi_{+,\sigma }\left(t\right)$ at a regular point $t_{0}$ (i.e., $t_{0}\in \left(t_{+,\sigma },2\right]=\mathrm{sdom}\;\phi_{+,\sigma }$) only once and transversally, from above to below. Otherwise, the root parabola $\phi_{\pm ,-,\sigma }\left(t\right)=\pm \sqrt{t-\phi_{+,\sigma }\left(t\right)}$ would have multiple branching points, contradicting Proposition 9. Singular points are isolated or at the boundary of smoothness domains (branching points), so the concept of transversal intersection do not apply to them.*

### 5.1. Symbolic Sequences

To study the superstable periodic orbits of $q_{t}$, it suffices to consider symbolic orbits. As an advantage, the results hold also under order-preserving conjugacies, as happens with ${\left.q_{t}\right|}_{\left[-2\mu ,2\mu \right]}$ and ${\left.f_{\mu }\right|}_{\left[0,1\right]}$ under the affine transformation (18). We come back to this point below.

Given a general orbit ${\left(q_{t}^{k}\left(x_{0}\right)\right)}_{k=0}^{\infty }=\left(x_{0},x_{1},\ldots ,x_{k},\ldots \right)$, the corresponding symbolic sequence $\Sigma =(\Sigma_{0},\Sigma_{1},\ldots ,\Sigma_{k},\ldots )$ is defined as follows:

$$\Sigma_{k}=\left\{\begin{array}{cc} - & \mathrm{if}\;x_{k}<0\\ C & \mathrm{if}\;x_{k}=0\\ + & \mathrm{if}\;x_{k}>0 \end{array}\right.$$

for k≥0. A symbolic sequence that corresponds to an actual orbit of $q_{t}$ for $t=t_{0}$ is called admissible (for $t=t_{0}$) and have to fulfill certain conditions [29].

Consider a superstable periodic orbit ${\left(0,x_{1},x_{2},\ldots ,x_{p-1}\right)}^{\infty }$ of prime period p≥2, so that $x_{k}\neq 0$ for k=1,…,p−1. For the time being, we drop the exponent “*∞*” and rearrange the cycle as $\left(x_{1},x_{2},\ldots ,x_{p-1},0\right)$ so that the first point is the critical value (also the greatest value) $q_{t}\left(0\right)=t>0$. In this case, $\Sigma_{k}\in \left\{+,-\right\}$ for 1≤k≤p−1, so that we will fittingly use σ’s instead of Σ’s and write the pertaining symbolic sequence as $\left(+,\sigma_{2},\ldots ,\sigma_{p-1},C\right)$. Therefore, by writing $\Sigma =(+,\sigma_{2},\ldots ,\sigma_{p-1},C)$ we do not need to specify that Σ is a superstable cycle of prime period *p*. The parameter values for which $q_{t}$ has superstable cycles are discrete because $q_{t}^{n}\left(0\right)=0$ is a polynomial equation in *t* for each *n* (see Remark 2); we will see below that there are infinitely many such values and that they accumulate at the right endpoint of the parametric interval, $t_{\max}=2$.

**Proposition** **11.** *Let $\Sigma =(+,\sigma_{2},\ldots ,\sigma_{p-1},C)$ be an admissible cycle for $t=t_{0}$. Then $t_{0}$ satisfies the equation*

(54) $$t_{0}=\phi_{+,-\sigma_{2},\ldots ,-\sigma_{p-1}}\left(t_{0}\right).$$

*Equivalently,*

(55) $$\phi_{\pm ,-,-\sigma_{2},\ldots ,-\sigma_{p-1}}\left(t_{0}\right)=0.$$

*Moreover, $t_{0}$ is a regular point of $\phi_{+,-\sigma_{2},\ldots ,-\sigma_{p-1}}\left(t_{0}\right)$, therefore $t_{0}$ is the branching point of the on-axis root parabola $\phi_{\pm ,-,-\sigma_{2},\ldots ,-\sigma_{p-1}}\left(t\right)$.*

**Proof.**  From (i) $x_{1}=q_{t_{0}}\left(0\right)=t_{0}$, (ii) $x_{k+1}=q_{t_{0}}\left(x_{k}\right)=t_{0}-x_{k}^{2}$, i.e.,

$$x_{k}=\sigma_{k}\sqrt{t_{0}-x_{k+1}}\;\;\mathrm{for}\;k=1,2,\ldots ,p-1,$$

and (iii) $x_{p}=0$, we obtain

$$t_{0}=\sqrt{t_{0}-x_{2}}=\sqrt{t_{0}-\sigma_{2}\sqrt{t_{0}-x_{3}}}=\ldots =\sqrt{t_{0}-\sigma_{2}\sqrt{t_{0}-\ldots -\sigma_{p-1}\sqrt{t_{0}}}}=\phi_{+,-\sigma_{2},\ldots ,-\sigma_{p-1}}\left(t_{0}\right).$$

Alternatively,

$$t_{0}=\phi_{+,-\sigma_{2},\ldots ,-\sigma_{p-1}}\left(t_{0}\right)\;\Leftrightarrow \;\;\pm \sqrt{t_{0}-\phi_{+,-\sigma_{2},\ldots ,-\sigma_{p-1}}\left(t_{0}\right)}\equiv \phi_{\pm ,-,-\sigma_{2},\ldots ,-\sigma_{p-1}}\left(t_{0}\right)=0.$$

Moreover, if $t_{0}$ is a singular point of $\phi_{+,-\sigma_{2},\ldots ,-\sigma_{p-1}}\left(t_{0}\right)$, then by Equation (33) and $t_{0}>0$, it holds $\phi_{-\sigma_{k+1},\ldots ,-\sigma_{p-1}}\left(t_{0}\right)=0$ for some 1≤k≤p−2, so that

$$t_{0}=\phi_{+,-\sigma_{2},\ldots ,-\sigma_{p-1}}\left(t_{0}\right)=\phi_{+,-\sigma_{2},\ldots ,-\sigma_{k}}\left(t_{0}\right).$$

It follows,

$$x_{k+1}=q_{t_{0}}^{k+1}\left(0\right)=q_{t_{0}}^{k}\left(t_{0}\right)=q_{t_{0}}^{k}\left(\phi_{+,-\sigma_{2},\ldots ,-\sigma_{k}}\left(t_{0}\right)\right)=0$$

by definition of root branches of rank *k*, which contradicts that $\Sigma_{k+1}\neq C$ (k+1≤p−1).

As explained in Remark 3, root branches have at regular points transversal intersections (if any) with the bisector. This implies that the branches of the root parabola $\phi_{\pm ,-,-\sigma_{2},\ldots ,-\sigma_{p-1}}\left(t\right)$ are defined in a neighborhood of $t_{0}$, therefore $t_{0}$ is a branching point of $\phi_{\pm ,-,-\sigma_{2},\ldots ,-\sigma_{p-1}}\left(t\right)$. □

By the same token, if there exists no solution of the equation $t=\phi_{+,-\sigma_{2},\ldots ,-\sigma_{p-1}}\left(t\right)$, then the cycle $\left(+,\sigma_{2},\ldots ,\sigma_{p-1},C\right)$ is not admissible. So, root branches and their bifurcation points, Equations (54) and (55), pop up as soon as one learns about superstable cycles. Next we prove the reverse implication of Proposition 11.

**Proposition** **12.** *If the bisector intersects transversally the root branch $\phi_{+,\sigma_{2},\ldots ,\sigma_{p-1}}\left(t\right)$ at $t_{0}$, then $\left(+,-\sigma_{2},\ldots ,-\sigma_{p-1},C\right)$ is an admissible cycle for $t=t_{0}$.*

**Proof.**  Suppose $q_{t_{0}}\left(0\right)=t_{0}=\phi_{+,\sigma_{2},\ldots ,\sigma_{p-1}}\left(t_{0}\right)$. Then, similarly to Equations (30) and (31),

$$\begin{array}{ccc} q_{t_{0}}^{2}\left(0\right) & = & q_{t_{0}}\left(\phi_{+,\sigma_{2},\ldots ,\sigma_{p-1}}\left(t_{0}\right)\right)=-\phi_{\sigma_{2},\ldots ,\sigma_{p-1}}\left(t_{0}\right)=\phi_{-\sigma_{2},\sigma_{3},\ldots ,\sigma_{p-1}}\left(t_{0}\right),\ldots \\ q_{t_{0}}^{k}\left(0\right) & = & q_{t_{0}}\left(\phi_{-\sigma_{k-1},\sigma_{k},\ldots ,\sigma_{p-1}}\left(t_{0}\right)\right)=-\phi_{\sigma_{k},\ldots ,\sigma_{p-1}}\left(t_{0}\right)=\phi_{-\sigma_{k},\sigma_{k+1},\ldots ,\sigma_{p-1}}\left(t_{0}\right) \end{array}$$

for k=3,…,p−1, and

$$q_{t_{0}}^{p}\left(0\right)=q_{t_{0}}\left(\phi_{-\sigma_{p-1}}\left(t_{0}\right)\right)=t_{0}-\phi_{-\sigma_{p-1}}{\left(t_{0}\right)}^{2}=t_{0}-{\left(-\sigma_{p-1}\sqrt{t_{0}}\right)}^{2}=0.$$

It follows that the points

$$\left(\phi_{+,\sigma_{2},\ldots ,\sigma_{p-1}}\left(t_{0}\right),\;\phi_{-\sigma_{2},\sigma_{3},\ldots ,\sigma_{p-1}}\left(t_{0}\right),\;\phi_{-\sigma_{3},\sigma_{4},\ldots ,\sigma_{p-1}}\left(t_{0}\right),\;\ldots ,\;\phi_{-\sigma_{p-1}}\left(t_{0}\right),\;0\right)$$

build a periodic orbit. Its symbolic sequence $\Sigma =(+,\Sigma_{2},\ldots ,\Sigma_{p-1},C)$ is determined by the signs of $\phi_{-\sigma_{k},\sigma_{k+1},\ldots ,\sigma_{p-1}}\left(t_{0}\right)$ for 2≤k≤p−1. Since $t_{0}$ is necessarily a regular point of $\phi_{+,\sigma_{2},\ldots ,\sigma_{p-1}}\left(t\right)$ (Remark 3), it holds $\phi_{-\sigma_{k},\sigma_{k+1},\ldots ,\sigma_{p-1}}\left(t_{0}\right)\neq 0$ for 2≤k≤p−1, and hence $\Sigma_{k}=-\sigma_{k}\in \left\{+,-\right\}.$ □

Table 2 lists the superstable cycles of $q_{t}$ of prime periods p=1,2,…6 (cycles written in abridged notation). The cycles of periods 2 and 3 are due to the transverse intersections of the bisector with $\phi_{+}\left(t\right)=\sqrt{t}$ at t=1 and with $\phi_{++}\left(t\right)=\sqrt{t+\sqrt{t}}$ at t≃1.7549. Figure 8 illustrates where the superstable cycles of prime periods 4 and 5 come from.

**Proposition** **13.** *The quadratic family has superstable cycles of arbitrary length.*

**Proof.**  First, $\mathrm{sdom}\;\phi_{{\left\{+\right\}}^{n}}=\left(0,2\right]$ for all n≥1, so all t>0 are regular points of $\phi_{{\left\{+\right\}}^{n}}\left(t\right)$. Second, the bisector and $\phi_{{\left\{+\right\}}^{n}}\left(t\right)$ always intersect transversally at a single point $t_{n}^{*}\in \left[1,2\right)$ because (i) $\phi_{{\left\{+\right\}}^{n}}\left(t\right)>t$ for 0<t<1, (ii) $\phi_{{\left\{+\right\}}^{n}}\left(2\right)<\phi_{{\left\{+\right\}}^{\infty }}\left(2\right)=2$ (see Equation (42)) and (iii) $\phi_{{\left\{+\right\}}^{n}}\left(t\right)$ is ∩-convex. Moreover, $t_{n}^{*}\to 2$ strictly monotonically as n→∞ because $\phi_{{\left\{+\right\}}^{n}}\left(t\right)<\phi_{{\left\{+\right\}}^{n+1}}\left(t\right)$ for all n≥1. The point $t_{n}^{*}$ is the branching point of the root parabola $\phi_{\pm ,-,{\left\{+\right\}}^{n-1}}\left(t\right)$, i.e., $t_{n}^{*}=$$t_{\pm ,-,{\left\{+\right\}}^{n-1}}$ in the notation of Section 3 and Section 4. □

According to Proposition 11, Equation (55), the parametric values of the superstable cycles of prime period p≥2 are the branching points of certain on-axis parabolas of the form $\phi_{\pm ,-,\alpha }\left(t\right)$ with $\left|\alpha \right|=p-2$. These parabolas originate precisely from the transversal intersections of the bisector with the root branches $\phi_{+,\alpha }\left(t\right)$ (if any). As in Section 3 and Section 4, those branching points are denoted by $t_{\pm ,-,\alpha }$ ($=t_{+,-,\alpha }=t_{-,-,\alpha }$). Therefore, the parameters of the superstable cycles can be ordered using the general ordering of the root branches, Proposition 7; alternatively, $t_{\pm ,-,\alpha }<t_{\pm ,-,\beta }$ if and only if $\phi_{+,-,\alpha }\left(2\right)>\phi_{+,-,\beta }\left(2\right)$. See Figure 9 for the on-axis parabolas of ranks 2–5; in case of equal branching points (e.g., $\phi_{\pm -}\left(t\right)$ and $\phi_{\pm ---}\left(t\right)$), only the lowest rank is shown because it corresponds to the prime period. The branching points are ordered as follows:

(56) $$t_{\pm -}<t_{\pm -+-}<t_{\pm -+--}<t_{\pm -+}<t_{\pm -++-}<t_{\pm -++}<t_{\pm -+++}$$

corresponding, respectively, to the superstable cycles

$$+C,\;\;+-+C,\;\;+-{\left\{+\right\}}^{2}C,\;\;+-C,\;+{\left\{-\right\}}^{2}+C,\;\;+{\left\{-\right\}}^{2}C,\;\;+{\left\{-\right\}}^{3}C$$

listed in Table 2 for prime periods 2–5. As shown in the proof of Proposition 13, the points $t_{\pm ,\alpha }$ (ordered as in Proposition 7) converge to 2 as $\left|\alpha \right|\to \infty$.

The superstable cycles of the quadratic family (and other three parametric families of transformations of the interval) were numerically studied in [30]. According to this paper, the number of superstable cycles of the quadratic family is as indicated in the following Table 3.

The parameter *t* of a superstable cycle $\left(+,\sigma_{2},\ldots ,\sigma_{n},C\right)$ can be numerically computed by means of the computational loop

$$t_{\nu +1}=\phi_{+,-\sigma_{2},\ldots ,-\sigma_{n}}\left(t_{\nu }\right),$$

ν=0,1,… until (i) $\left|t_{\nu +1}-\phi_{+,-\sigma_{2},\ldots ,-\sigma_{n}}\left(t_{\nu }\right)\right|<\varepsilon$, where ε>0 is the desired precision, or (ii) a prefixed maximum number of loops $\nu_{\max}$ is reached, flagging that the convergence $t_{\nu }\to t_{+,-\sigma_{2},\ldots ,-\sigma_{n}}$ is too slow.

**Theorem** **3.** *(a) A symbolic cycle $\Sigma =(+,\sigma_{2},\ldots ,\sigma_{s-1},C)$ can be admissible only for one value of t. (b) If Σ is admissible for $t=t_{1}$ and $\Sigma^{\prime }=\left(+,\rho_{2},\ldots ,\rho_{r-1},C\right)\neq \Sigma$ is admissible for $t=t_{2}$, then $t_{1}\neq t_{2}$.*

**Proof.**  (a) Suppose $\left(+,\sigma_{2},\ldots ,\sigma_{s-1},C\right)$ is admissible for two different parametric values $t_{1}$ and $t_{2}$. By Proposition 11, $t_{1}$ and $t_{2}$ are then two branching points of $\phi_{+,-,-\sigma_{2},\ldots ,-\sigma_{s-1}}\left(t\right)$, which contradicts Proposition 9.

(b) Suppose by contradiction that $t_{1}=t_{2}=t_{0}$. By Proposition 11,

$$t_{0}=\phi_{+,-\sigma_{2},\ldots ,-\sigma_{s-1}}\left(t_{0}\right)=\phi_{+,-\rho_{2},\ldots ,-\rho_{r-1}}\left(t_{0}\right),$$

where $t_{0}$ is a regular point. On the other hand, according to Proposition 7, root functions can coincide only at singular points. □

**Remark** **4.** *The main ingredient in the proof of Theorem 3 is the fact that $\mathrm{sdom}\;\phi_{\sigma }$ is a half-interval $\left(t_{\sigma },2\right]$ (Proposition 9), from which Milnor’s Monotonicity Conjecture (Theorem 2) was derived. Reciprocally, from Theorem 3 it follows that the bisector can transversally intersect a positive root branch $\phi_{+,\sigma }\left(t\right)$ only once. In turn, it recursively follows from this that the simplicity domains of the root branches are half-intervals and, hence, Milnor’s Monotonicity Conjecture.*

Two maps of the interval f(x) and g(y) are called combinatorially equivalent if they are conjugate via an order-preserving transformation φ(x). For instance, $q_{t}\left(x\right)$ and $cq_{1/c}\left(y\right)=1-cy^{2}$ are combinatorially equivalent via $\varphi \left(x\right)=\frac{1}{t}x$ and c=t, whereas $q_{t}\left(x\right)$ and $-q_{-c}\left(y\right)=c+y^{2}$ are conjugate via φ(x)=−x and c=−t, but they are not combinatorially equivalent because φ(x) reverses the order in this case. It is plain that combinatorially equivalent maps have the same symbolic sequences for corresponding initial conditions $x_{0}$ and $\varphi (x_{0})$**.**

**Theorem** **4** (Thurston’s Rigidity [15]). *Consider $q_{t_{1}}$ and $q_{t_{2}}$ for which their critical points c=0 have finite orbits O and $O^{\prime }$. If $q_{t_{1}}$ and $q_{t_{2}}$ are combinatorially equivalent, then $t_{1}=t_{2}$.*

**Proof.**  Suppose that $q_{t_{1}}$ and $q_{t_{2}}$ are combinatorially equivalent via an order-preserving conjugacy φ. Then, the symbolic sequence Σ of O and the symbolic sequence $\Sigma^{\prime }$ of $\varphi \left(O\right)=O^{\prime }$ are equal. Apply now Theorem 3(a) to conclude $t_{1}=t_{2}$. □

As mentioned in the Introduction, Thurston’s Rigidity implies Milnor’s Monotonicity Conjecture for the quadratic maps [15]. In Remark 4 we sketched how this derivation could be done using Theorem 3, which is a sort of symbolic version of Thurston’s Rigidity.

### 5.2. Dark Lines and the Misiurewicz Points

To wrap up our excursion into the superstable cycles of the quadratic family, let us remind that the “dark lines” in the bifurcation diagram (Figure 3) that go through the chaotic regions or build their boundaries are determined by the superstable periodic orbits. To briefly study those dark lines, we resort again to the polynomials $P_{n}\left(t\right)\equiv q_{t}^{n}\left(0\right)$ introduced in Equations (47) and (49).

We have already discussed in Section 5.1 how to pinpoint superstable cycles ${\left(0,P_{1}\left(t\right),\ldots ,P_{p-1}\left(t\right)\right)}^{\infty }$ in the parametric interval using symbolic sequences and root branches. More generally, consider orbits of 0 that are eventually periodic, that is:

(57) $${\left(P_{n}\left(t\right)\right)}_{n=0}^{\infty }=\left(0,t,P_{2}\left(t\right),\ldots ,P_{h-1}\left(t\right),{\left(P_{h}\left(t\right),P_{h+1}\left(t\right),\ldots ,P_{h+T-1}\left(t\right)\right)}^{\infty }\right).$$

Such parametric values are called Misiurewicz points [31] and denoted as $M_{h,T}$, where we assume that h≥1 is the minimal length of the preperiodic ”tail” (the preperiod) and T≥1 is the prime period of the periodic cycle. Therefore, if $M_{h,T}$ is a Misiurewicz point, then

(58) $$P_{h}\left(M_{h,T}\right)=P_{h+T}\left(M_{h,T}\right)=P_{h+2T}\left(M_{h,T}\right)=\ldots ,$$

so that the curves $P_{h+kT}\left(t\right)$, k≥0, meet at $t=M_{h,T}$ in the (t,x)-plane.

For example,

(59) $${\left(P_{n}\left(2\right)\right)}_{n=0}^{\infty }=\left(0,2,{\left(-2\right)}^{\infty }\right),$$

i.e., the orbit of 0 hits a (repelling) fixed point after only two iterations. Comparison of Equations (59) and (57) shows that $2=M_{2,1}$, therefore, all curves $P_{n}\left(t\right)$ with n≥2 meet at t=2 (see Equation (51)).

The graphs of $P_{0}\left(t\right),\ldots ,P_{7}\left(t\right)$ are shown in Figure 10. As a first observation, one can recognize the main features of the chaotic bands in the bifurcation diagram of the quadratic family, in particular band merging. We also see that the curves $x=P_{n}\left(t\right)$ intersect transversally or tangentially; all these intersections are related to important aspects of the dynamic. Chaos bands merge where those curves intersect transversally, while periodic windows open where they intersect tangentially the upper and lower edges. Moreover, the functions $P_{n}\left(t\right)$ intersect the *t*-axis precisely at the parameter values for which 0 is periodic:

$$P_{n}\left(t_{0}\right)\equiv q_{t_{0}}^{n}\left(0\right)=0\;\;\Leftrightarrow \;\;\phi_{\sigma_{1},\ldots ,\sigma_{n}}\left(t_{0}\right)=0.$$

Besides $P_{n}\left(0\right)=0$ for all n≥0 (Equation (50)) and $P_{n}\left(1\right)=0$ for all n≥1 (Equation (47)), the following zeros of $P_{n}\left(t\right)$ can be read in Figure 9: $P_{3}\left(t\right)=0$ at t≃1.7549; $P_{4}\left(t\right)=0$ at t=1.3107 and 1.9408; and $P_{5}\left(t\right)=0$ at t=1.6254, 1.8607 and 1.9854.

As way of illustration, we will calculate $M_{3,1}$, the perhaps most prominent Misiurewicz point in Figure 10, which corresponds to the merging of the two chaotic bands into a single band. By Equation (58) with h=3 and T=1,

$$P_{3}\left(M_{3,1}\right)=P_{4}\left(M_{3,1}\right)=P_{5}\left(M_{3,1}\right)=\ldots$$

From $P_{3}\left(M_{3,1}\right)=P_{4}\left(M_{3,1}\right)$ and Equation (49) if follows that $M_{3,1}$ is the unique real solution in (0,2) of the equation

$$4-6t+6t^{2}-4t^{3}+t^{4}=0,$$

namely, $M_{3,1}=1.5436890\ldots$ For more in-depth information, the interested reader is referred to [32,33].

Among the many remarkable properties of the Misiurewicz points, we highlight only the following two: (i) the periodic cycle ${\left(P_{h}\left(M_{h,T}\right),P_{h+1}\left(M_{h,T}\right),\ldots ,P_{h+T-1}\left(M_{h,T}\right)\right)}^{\infty }$ in Equation (57) is repelling [11], and (ii) $q_{t}\left(x\right)$ has an absolutely continuous invariant measure for each $t=M_{h,T}$ [34,35].

## 6. Conclusions

In the previous sections we have revisited two classical topics of the continuous dynamics of interval maps: entropy monotonicity (Section 4) and superstable cycles (Section 5) for the quadratic family $q_{t}\left(x\right)$ (Section 3). The novelty consists in the starting point: we use Equation (52) for $h(q_{t})$, the topological entropy of $q_{t}$, where $s_{n}\left(t\right)$ is the number of transversal intersections of the polynomial curves $q_{t}^{n}\left(x\right)$ with the *x*-axis. Equation (52) and several numerical schemes for its computation were derived in [7,8,9]. This approach leads directly to the root functions ($\phi_{\sigma }\left(t\right)$), bifurcation points ($t_{\sigma }$) and smoothness domains ($\mathrm{sdom}\;\phi_{\sigma }$) studied in Section 3.1 and Section 3.2. It is precisely the structure of the smoothness domains, $\mathrm{sdom}\;\phi_{\sigma }=\left(t_{\sigma },2\right]$ (Proposition 9), which implies that $s_{n}\left(t\right)$ is a nondecreasing staircase function for each n≥1 (Theorem 1) and, in turn, that the function $t\mapsto h(q_{t})$ is monotone (Theorem 2). Unlike existing proofs [3,11,12,13], Theorem 2 proves Milnor’s Monotonicity Conjecture via real analysis. This also shows that the transversal intersections of a multimodal map and its iterates with the critical lines is a useful tool in one-dimensional dynamics. Section 2.2 and Section 4 contains further details on Milnor’s Monotonicity Conjecture and its generalization to multimodal maps.

In Section 5.1 we derived some basic results on the superstable cycles of the quadratic family, in particular Theorem 3, which is a sort of symbolic version of Thurston Rigidity (Theorem 4). The commonalities between entropy monotonicity and the superstable cycles of the quadratic maps go beyond the techniques used, namely, root branches, bifurcation points, transversality, and a geometrical language. There is also a flow of ideas in both directions. We started with the topological entropy and worked our way towards the superstable cycles, but the other direction works too, although we only indicated this possibility in Remark 4. We also made a brief excursion into the preperiodic orbits of the critical point in Section 5.2 (Misiurewicz points). In conclusion, both topics complement and intertwine in remarkable ways, as well as being interesting on their own.

## Figures and Tables

**Figure 1:**
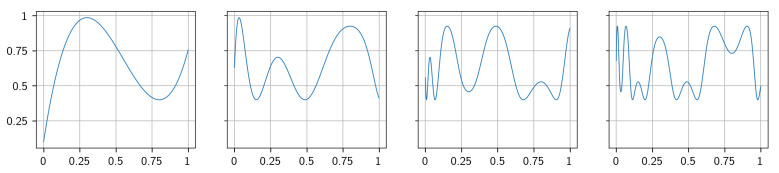
Graphs of *f*, $f^{2}$, $f^{3}$, and $f^{4}$ for the bimodal map (7). Reproduced from [7].

**Figure 2:**
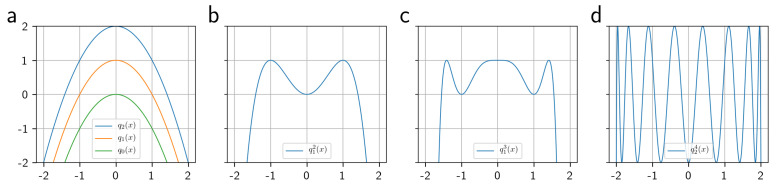
(**a**) Bottom to top: $q_{0}\left(x\right)$, $q_{1}\left(x\right)$, $q_{2}\left(x\right)$. (**b**) Graph of $q_{1}^{2}\left(x\right)$. (**c**) Graph of $q_{1}^{3}\left(x\right)$. (**d**) Graph of $q_{2}^{4}\left(x\right)$.

**Figure 3:**
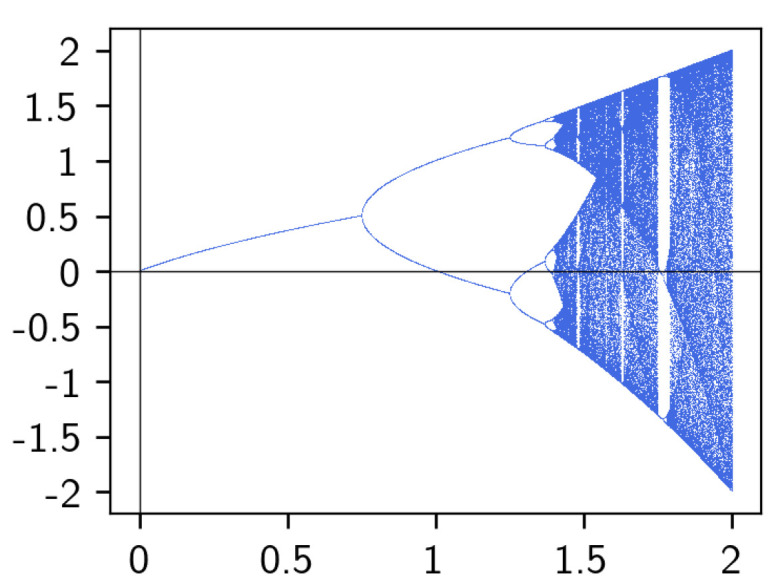
Bifurcation diagram of $q_{t}\left(x\right)$, 0≤t≤2.

**Figure 4:**
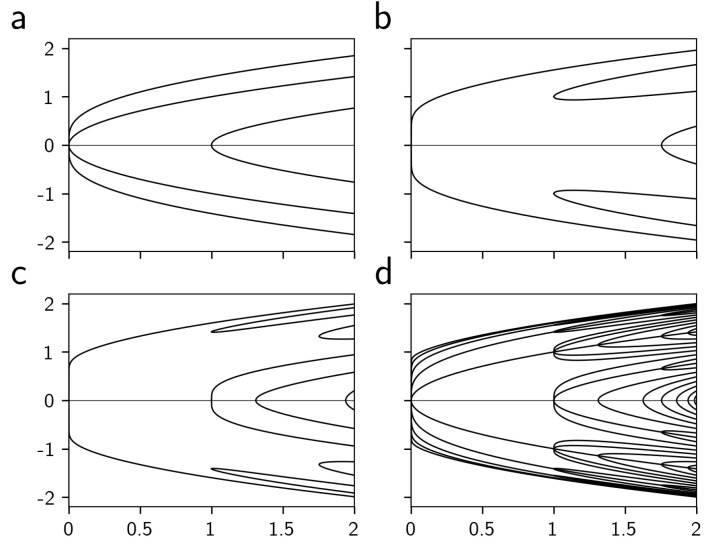
Root branches of ranks 1 to 5; since $\phi_{-,\sigma }\left(t\right)=-\phi_{+,\sigma }\left(t\right)$, only positive branches (upper half-plane) are specified next (top to bottom). (**a**) Ranks 1 and 2: $\phi_{++}$, $\phi_{+}$, and $\phi_{+-}$. (**b**) Rank 3: $\phi_{+++}$, $\phi_{+\pm -}$, and $\phi_{+-+}$. (**c**) Rank 4: $\phi_{++++}$, $\phi_{++\pm -}$, $\phi_{+\pm -+}$, $\phi_{+---}$, $\phi_{+-+-}$, and $\phi_{+-++}$. (**d**) Joint plot of the root branches of ranks 1 to 5; positive branches of rank 5: $\phi_{+++++}$, $\phi_{+++\pm -}$, $\phi_{++\pm -+}$, $\phi_{++---}$, $\phi_{++-+-}$, $\phi_{++-++}$, $\phi_{+--++}$, $\phi_{+--+-}$, $\phi_{+----}$, $\phi_{+-\mp -+}$, $\phi_{+-+--}$, $\phi_{+-++-}$, and $\phi_{+-+++}$.

**Figure 5:**
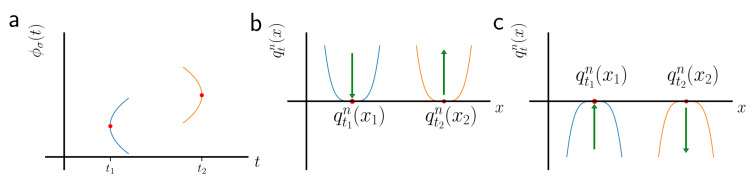
(**a**) Root branches not connected to the boundary t=2. As a new feature, the bifurcation at $t_{2}$ opens to the left. (**b**) A first possibility in state space for local extrema of $q_{t}^{n}\left(x\right)$ to produce the bifurcations in panel (**a**). (**c**) A second possibility in state space for local extrema of $q_{t}^{n}\left(x\right)$ to produce the bifurcations in panel (**a**).

**Figure 6:**
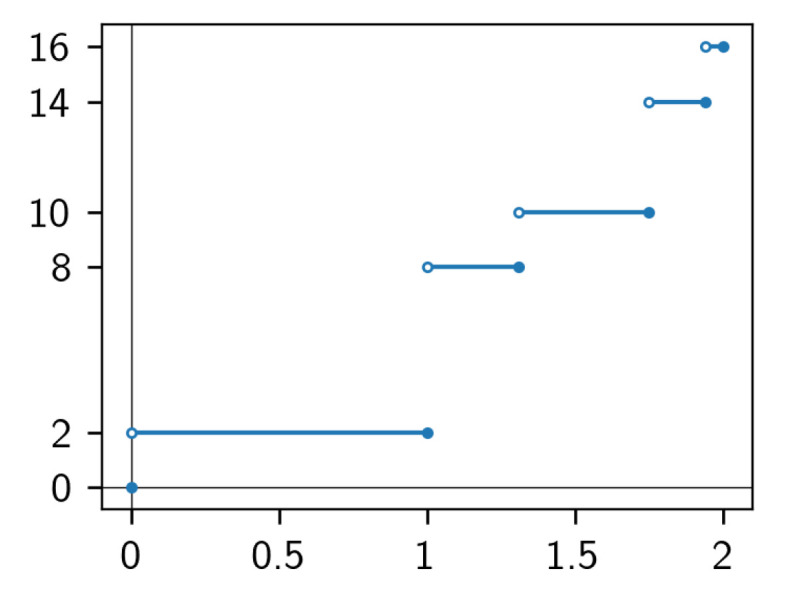
The function $s_{4}\left(t\right)$ for the quadratic family. Jumps occur at the branching points $t_{++++}=0$, $t_{+---}=t_{++--}=t_{+++-}=1$, $t_{+-+-}≃1.3107$, $t_{+--+}=t_{++-+}≃1.7549$, and $t_{+-++}≃1.9408$.

**Figure 7:**
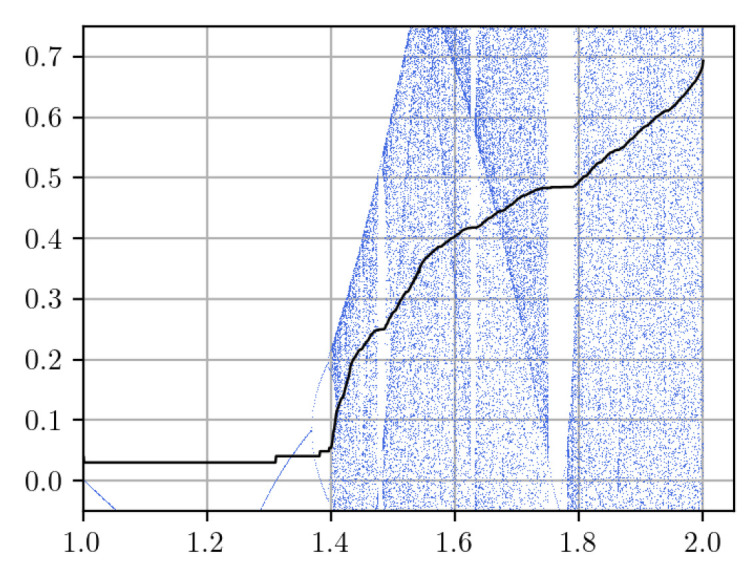
Topological entropy of $q_{t}$ using the Formula (52) with the logarithm to the base *e*. The topological entropy was plotted on the bifurcation diagram for a better understanding of its characteristics.

**Figure 8:**
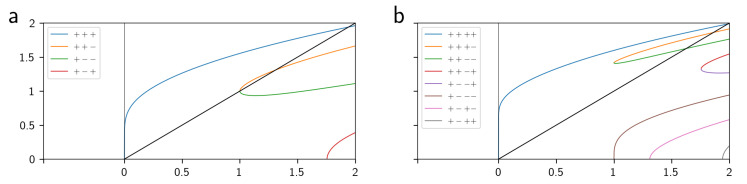
(**a**) Non-transverse intersections (“T-crossings”) of the bisector with $\phi_{++-}\left(t\right)$ and $\phi_{+--}\left(t\right)$ at t=1, and transverse intersections of the bisector with (bottom to top) $\phi_{++-}\left(t\right)$ at t≃1.3107 and $\phi_{+++}\left(t\right)$ at t≃1.9408. Proposition 12 entails that $q_{1.3107\ldots }$ has the cycle (+,−,+,C) and $q_{1.9408\ldots }$ the cycle (+,−,−,C), whereas $q_{1}$ has the cycle (+,C). (**b**) Regular intersections of the bisector with (bottom to top) $\phi_{++--}\left(t\right)$ at t≃1.6254, $\phi_{+++-}\left(t\right)$ at t≃1.8607, and $\phi_{++++}\left(t\right)$ at t≃1.9854. Proposition 12 entails that $q_{1.6254\ldots }$, $q_{1.8607\ldots }$ and $q_{1.9854\ldots }$ have the cycles (+,−,+,+,C), (+,−,−,+,C), and (+,−,−,−,C), respectively.

**Figure 9:**
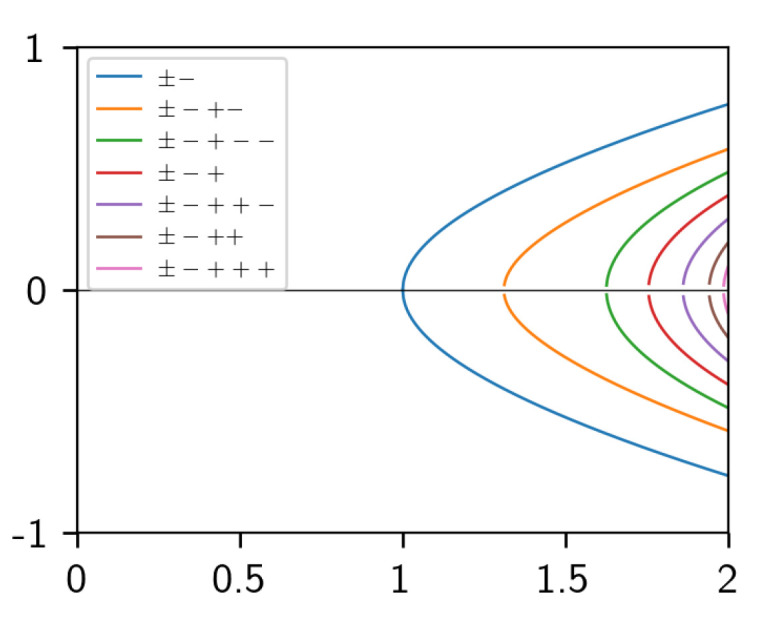
On-axis root parabolas of ranks 2–5. In case of parabolas with coinciding branching points, only the parabola with the lowest rank (corresponding to the prime period of the superstable cycle) is shown. The branching points (see Equation (56)) are: $t_{\pm -}=1$, $t_{\pm -+-}≃1.3107$, $t_{\pm -+--}≃1.6254$, $t_{\pm -+}≃1.7549$, $t_{\pm -++-}≃1.8607$, $t_{\pm -++}≃1.9408$, and $t_{\pm -+++}≃1.9854$. The first four parameter values (periods 2, 4, 5 and 3) are clearly visible in the bifurcation diagram, Figure 3, at the intersection of periodic attractors with the axis x=0.

**Figure 10:**
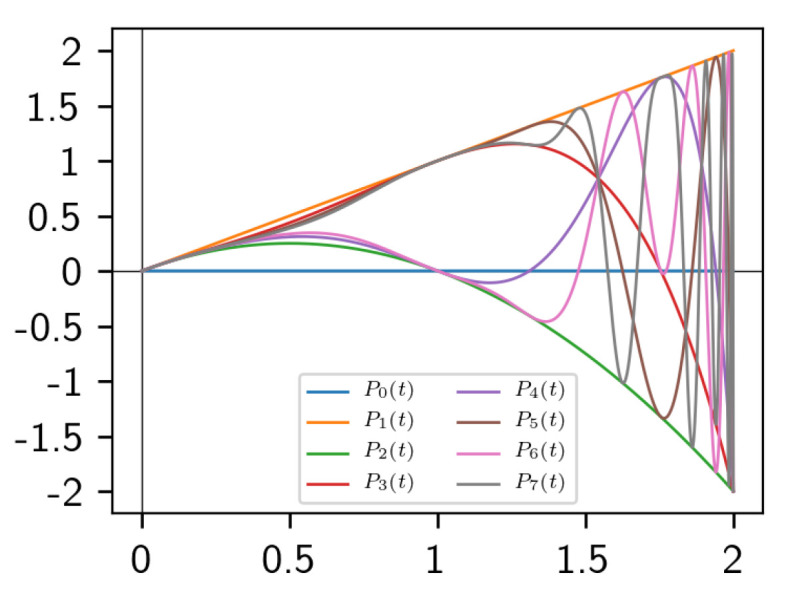
The polynomials $P_{n}\left(t\right):=q_{t}^{n}\left(0\right)$ for 0≤n≤7.

**Table 1:** Root parabolas of ranks 1 to 4.

Root Parabolas	$\mathrm{Sdom}\;\phi_{\sigma }$
$\phi_{\pm },\phi_{\pm +},\phi_{\pm ++},\phi_{\pm +++}$	(0,2]
$\phi_{\pm -},\phi_{+\pm -},\phi_{-\pm -},\phi_{++\pm -},\phi_{-+\pm -},\phi_{\pm ---}$	(1,2]
$\phi_{\pm -+-}$	(1.3107…,2]
$\phi_{\pm -+},\phi_{+\pm -+},\phi_{-\pm -+}$	(1.7549…,2]
$\phi_{\pm -++}$	(1.9408…,2]

**Table 2:** Superstable cycles of periods 1 to 6.

Period	Superstable Cycles
1	*C*
2	+C
3	+−C
4	$+-+C,\;+{\left\{-\right\}}^{2}C$
5	$+-{\left\{+\right\}}^{2}C,$ $+{\left\{-\right\}}^{2}+C,$ $+{\left\{-\right\}}^{3}C$
6	$+-{\left\{+\right\}}^{3}C,\;+{\left\{-\right\}}^{2}+-C,\;+{\left\{-\right\}}^{2}{\left\{+\right\}}^{2}C,\;+{\left\{-\right\}}^{3}+C,\;+{\left\{-\right\}}^{4}C$

**Table 3:** Number of superstable cycles of the quadratic family.

Period	2	3	4	5	6	7	8	9	10	11	12	13	14	15
**# sup. cycles**	1	1	2	3	5	9	16	28	51	93	170	315	585	1091
